# CHOP: haplotype-aware path indexing in population graphs

**DOI:** 10.1186/s13059-020-01963-y

**Published:** 2020-03-11

**Authors:** Tom Mokveld, Jasper Linthorst, Zaid Al-Ars, Henne Holstege, Marcel Reinders

**Affiliations:** 1 Delft Bioinformatics Lab, Delft University of Technology, Van Mourik Broekmanweg 6, Delft, 2628 XE The Netherlands; 2 Computer Engineering, Delft University of Technology, Mekelweg 4, Delft, 2628 CD The Netherlands; 3 Department of Clinical Genetics, VU University Medical Center, Van der Boechorststraat 7, Amsterdam, 1081 BT The Netherlands

**Keywords:** Graph-based reference genomes, Read alignment, Haplotype-aware graph indexes

## Abstract

The practical use of graph-based reference genomes depends on the ability to align reads to them. Performing substring queries to paths through these graphs lies at the core of this task. The combination of increasing pattern length and encoded variations inevitably leads to a combinatorial explosion of the search space. Instead of heuristic filtering or pruning steps to reduce the complexity, we propose CHOP, a method that constrains the search space by exploiting haplotype information, bounding the search space to the number of haplotypes so that a combinatorial explosion is prevented. We show that CHOP can be applied to large and complex datasets, by applying it on a graph-based representation of the human genome encoding all 80 million variants reported by the 1000 Genomes Project.

## Introduction

Pangenomes and their graphical representations have become widespread in the domain of sequencing analysis [1]. Part of this adoption is driven by the increased characterization of within-species genomic diversity. For instance, recent versions of the human reference genome (GRCh37 and up) include sequences that represent highly polymorphic regions in the human population [2].

A pangenome can be constructed by integrating known variants in the linear reference genome. This way, a pangenome can incorporate sequence diversity in ways that a typical linear reference genome cannot. For example aligning reads to a linear reference genome can lead to an over-representation of the reference allele. This effect, known as reference allele bias, influences highly polymorphic regions and/or regions that are absent from the reference [3, 4]. By integrating variants into the alignment process, this bias can be reduced [5–7]. As a consequence, variant calling can be improved, with fewer erroneous variants induced by misalignments around indels and fewer missed variants [8]. An intuitive representation for pangenomes is graph data structures, which are often referred to as population graphs [1, 9]. Population graphs can be understood as compressed representations of multiple genomes, with sequence (in some cases of both complements) generally represented on the nodes. These nodes are in turn connected by (bi)-directed edges, such that the full sequence of any genome used to construct the graph can be determined by a specific path traversal through the graph. Alternatively, an arbitrary path traversal will yield a mixture of genomes.

A key application of reference genomes is read alignment. Most of the linear reference read aligners follow a seed-and-extent paradigm, wherein exact matching substrings (seeds) between the read and a reference are used to constrain a local alignment. To efficiently search for exactly matching substrings (seeding), indexing data structures are used. The construction of these indexes generally relies on one of two methods: hashing-based indexing, which can either be *k*-mer-based where all substrings of length *k* of the reference are stored in a hash map along with their positions [10, 11]; fingerprinting-based hashing which allows for finding candidate alignment positions as a nearest neighbor search approximating the Jaccard set similarity using MinHash [12, 13]; and sorting-based methods such as the Burrows-Wheeler transform (BWT) [14, 15], where the reference sequence is transformed into a self-index that supports the lookup of exact-matching substrings of arbitrary length.

Existing indexing methods can be extended to population graphs, though this is challenging. Graphs can encode a variable number of genomes, which comes with an exponential growth of the number of paths in the graph as more variation is integrated. Arbitrary length sequence indexing is therefore highly challenging, and indexing must generally be limited to shorter substrings to minimize combinatorial growth of the index. Additionally, sorting-based indexing methods that rely on suffix determination and ordering are often infeasible in graphs since there will be multiple valid node orderings.

Several approaches have been developed that perform read alignment onto population graphs using indexes that report all *k*-length paths in the graph. Early examples of this include the following: GenomeMapper [16], which builds a joint *k*-mer hash map combining a collection of genomes to lookup seeds and subsequently align reads, using banded dynamic programming; BWBBLE [17], which linearizes the population graph using IUPAC encoding for SNPs and describes indels with flanking sequences as alternate contigs, after which it applies the BWT for indexing. In Satya et al. [18], they generate an enhanced reference genome from HapMap SNP-chip calls, wherein variants are encoded in read length segments used as alternative alignment targets alongside the reference genome. These methods are, however, orders of magnitude slower than linear reference genome aligners or restricted to only small genomes. For instance, BWBBLE computes four times more suffix array intervals due to the expanded IUPAC alphabet. Moreover, these methods suffer from exponential growth in index space when variation density increases.

Increased scalability of population graph alignment has recently been demonstrated with Graphtyper [19], GraphAligner (designed for long reads) [20], the variation graph toolkit vg [21], and HiSat2 [22]. Graphtyper does so by first aligning reads to a linear reference sequence using BWA (as such, there remains implicit reference bias), after which a graph-based alignment is performed on a much smaller set of unaligned or partially aligned reads. For this graph alignment, it uses a *k*-mer hash map of the population graph, wherein exponential growth in variation-dense regions is reduced through the removal of *k*-mers that overlap too many alternative sequences. GraphAligner utilizes minimizers, maximal unique matches, or maximal exact matches to seed read to graph alignments. Seeds are extended and aligned using a bitvector-banded dynamic programming algorithm for both acyclic and arbitrary cyclic graphs. The vg toolkit provides general solutions for working with population graphs. To efficiently query substrings, it utilizes GCSA2 indexing [23], an extension of the BWT for population graphs, which supports exact query lengths of up to 256 bp. Reads are aligned to graphs using a seed-and-extend strategy, returning subgraphs of the population graph to which reads are subsequently aligned using partial order alignment, a generalization of pairwise sequence alignment for directed acyclic graphs [24]. HiSat2 generates a global graph FM index along with multiple smaller region-specific graph FM indexes. This index builds upon the GCSA [25], the precursor of the GCSA2 index utilized by vg.

Graphtyper and vg index all possible paths in a population graph, in which they also cover complex regions where the variation is dense. To deal with this, heuristics are utilized to prevent exponential growth. Such heuristics either remove *k*-mers that cross over more than a predefined number of edges or mask out subgraphs shorter than a set number of bases. Techniques as these do prevent exponential growth, but can completely remove complex regions in the graph, resulting in a loss of sensitivity in alignment. Furthermore, they contradict one of the main aims of population graphs, namely to address sequence variation in regions that are inaccessible through the application of a single reference sequence. Similarly, HiSat2 filters rare variants from the graph, effectively reducing the complexity of the graph, but also at the cost of addressing less sequence variation. An alternative solution that does not exclude complex regions, would be to constrain indexing by haplotype, so only *k*-mers observed in the linear genomes are encoded in the index. While the above heuristics are also used in vg, the authors of vg have recently also proposed the use of haplotyping. In vg, such haplotyping is facilitated using the GBWT [26–28]. The GBWT is a graph extension of the positional Burrows-Wheeler transform [29] that can store the haplotypes of samples as paths in the graph, allowing for haplotype-constrained read alignment. However, note that the GBWT index is build alongside the GCSA2 index which will still require an evaluation of all *k*-paths in the graph (that will ultimately be pruned using the GBWT index). So, although vg+GBWT indeed incorporates haplotype constraints when aligning reads, during indexing, the complexity is still dictated by the GCSA2 indexing which explores all *k*-paths and thus grows exponentially with the amount of variations.

We present CHOP, an alternative path indexer for population graphs, that utilizes haplotype-level information to constrain the process of path indexing without the need for heuristic filtering or pruning steps. This constraint eliminates the need to evaluate all *k*-paths and avoids the exponential growth in *k*-paths that other methods run into. CHOP decomposes the graph into a set of linear sequences, similarly as in [18, 30], such that reads can be aligned by long established aligners, such as BWA or Bowtie2 [31, 32], which can then be followed up by typical downstream analysis. We show that the alignment performance of BWA when using CHOP performs comparably to vg, but that with CHOP, alignment is faster and can scale more effectively to graphs build from human genomes with variation data of the 1000 Genomes Project [33].

In summary, the contributions of our work are as follows: (1) CHOP decomposes a population graph into mappable sequences representing all observed haplotypes with which the population graph was built; (2) the haplotype constraint is implemented in such a way that any exponential exploration of the graph is avoided, eliminating the need to filter or prune the graph in any way so that the complexity is bounded by the number of encoded haplotypes (instead of the number of variants or *k*-paths in the graph); (3) the decomposition of the graph can be done in a time- and memory-efficient way, keeping indexing time and memory low; and (4) by decomposing the graph into mappable sequences, it is possible to use standard aligners to map reads, with which one can benefit from fast alignment times as well as buildup experience with parameter settings of these aligners.

## Results

**Fig. 1:**
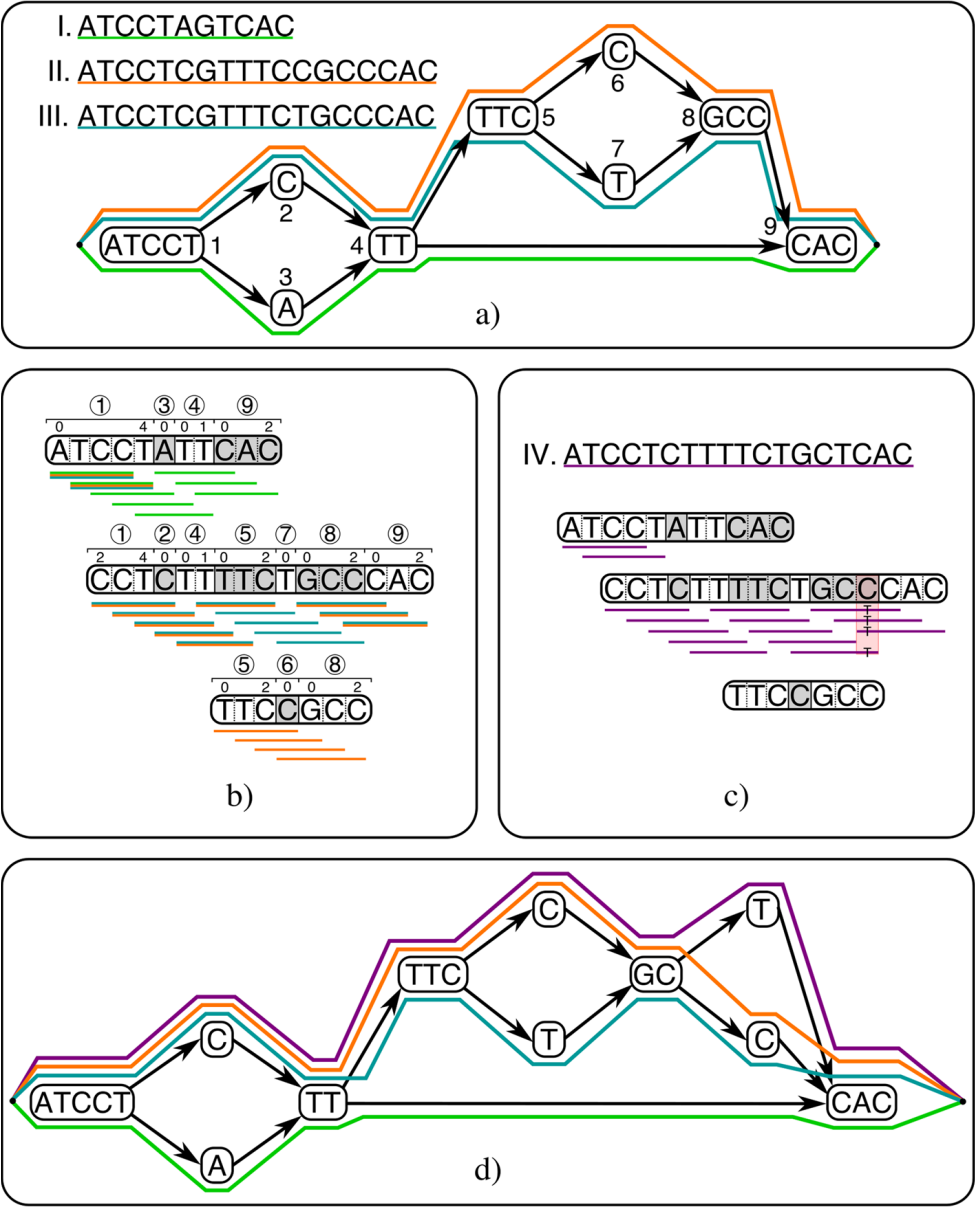
Schematic overview of how CHOP aligns reads to a population graph. **a** As input, CHOP accepts a graphical representation of three distinct haplotypes (I, II, III). Colored paths through the graph identify underlying haplotypes. **b** CHOP decomposes the graph into a null graph (a graph devoid of edges) for substrings of length 4 (Additional file 1: Figure S1 gives the full details about the decomposition). The obtained null graph contains three nodes, and the sequence that is defined on these nodes covers all substrings of length 4 which occur in the haplotypes encoded in the graph. Annotations above each node refer to intervals within nodes of the input graph. **c** The reads (with length 4) from a new haplotype (IV) can be aligned to the null graph; consequently, a mismatch can be called from the read pileup. **d** Through the attached interval definitions that are assigned to the null graph, the novel variant can be positioned on node 8 of the original graph. Incorporating this variant results in a new graph

Throughout, we consider population graphs constructed from variations called per sample (haplotype) with respect to a linear reference genome. These variations are encoded in the graph such that nodes represent sequences and edges represent observed consecutive sequences (the “Methods” section). CHOP facilitates read-to-graph alignment, which is presented in detail in the “Methods” section. Briefly, CHOP transforms a population graph into a null graph (a graph devoid of edges) by a series of operations consisting of three steps: collapse, extend, and duplicate, such that nodes in the null graph contain every substring of length *k* originating from the encoded original haplotypes in the population graph. Established aligners (here we have used BWA) can then be used to align reads to these null graph node sequences. Subsequently, these alignments can be projected back onto the population graph, given that the mapping of the node sequences in the null graph is known in the population graph (Fig. 1).

### Evaluation graph alignment

To evaluate CHOP, and its applicability in population graph alignment, we first performed tests on *Mycobacterium tuberculosis* (MTB) using the read aligner BWA-0.7.15-MEM [31]. MTB represents a good model for population graphs, given the high accuracy of available assemblies, the tractable genome size (4.4 Mb), and the limited degree of variation. Four hundred one variant call sets (VCF files) from different MTB strains (samples) were obtained from the KRITH1 and KRITH2 datasets [34, 35]. Variants were called with respect to the reference genome H37Rv, using Pilon-1.22 [36], and were filtered to exclude low-quality variants. For graph construction, we employed a leave-one-out strategy, wherein 1 sample was removed from the VCF file containing all 401 samples. The read set of the removed sample was subsequently used for graph alignment. This was repeated with 10 randomly selected samples. Corresponding single-end read sets were obtained from EBI-ENA (Additional file 1: Section 2). To investigate how the introduction of more variation influences the graph alignment, we progressively incorporated more samples (from the complete set) into the constructed graphs, with up to 17,500 variants in the 400 sample graph (rate of variation growth is shown in Additional file 1: Figure S2).

As the ground truth of genomic positions in the read set data is unknown, we evaluated alignments based on the following criteria: number of mismatches, insertions, deletions, clipped bases, unaligned reads, and perfectly aligned reads (definitions in Additional file 1: Section 4). These criteria allowed us to inspect the behavior of different read aligners. In order to avoid bias induced by multiple possible alignments for a single read, we only considered primary alignments.

To evaluate our haplotype-based approach, we compared CHOP to vg-1.12.1 with haplotyping (denoted as vg+GBWT) and without. The vg toolkit provides general solutions for population graphs which include graph construction, indexing, and read alignments. CHOP was set to report 101-length haplotype paths (equivalent to the read length) and used default parameters with BWA-MEM. vg was set to index all 104-length paths (*k*=13, 3 doubling steps), to most closely reflect the settings of CHOP.

Because CHOP uses BWA as an aligner while vg has its own internal aligner, differences based on the aligner and not the indexing algorithm may occur. To understand the aligner- and parameter-induced differences, we first summarized the results of the ten hold-out samples on the linear reference genome, shown in Table 1 for BWA and vg. Both aligners resulted in nearly the same number of perfectly aligned reads. However, alignments with vg resulted in fewer unaligned reads (− 22.30*%*) and more mismatches (+ 4.01*%*) than BWA. We attribute this difference to an increase in sensitivity by which vg aligns reads. This is reflected by the increase in clipped bases (+ 22.79*%*), inserted bases (+ 29.36*%*), and deleted bases (+ 34.53*%*), which allows vg to align shorter read fragments.

**Table 1:** Mean of alignment results across all 10 hold-out sample alignments to (1) the reference genome H37Rv (H37Rv columns) and (2) the 400 MTB genomes graph (graph columns) for both CHOP/BWA and vg with and without haplotyping to align the reads (note that when aligning only to H37Rv, CHOP is not used)

	All TB hold-out samples, read length = 101						
Alignment criteria	BWA	CHOP/BWA	vg	vg	vg + GBWT	HiSat2	HiSat2
	H37RV	Graph (*n* = 400)	H37RV	Graph (*n* = 400)	Graph (*n* = 400)	H37RV	Graph (*n* = 400)
Reads aligned	6,160,920	6,162,033 (+ 0.018%)	6,241,270	6,245,907 (+ 0.074%)	6,244,004 (+ 0.044%)	5,536,194	5,489,149 (− 0.850%)
Reads unaligned	360,236	359,123 (− 0.309%)	279,886	275,249 (− 1.657%)	277,152 (− 0.977%)	984,962	1,032,007 (+ 4.776%)
Reads perfectly aligned	4,048,774	4,142,052 (+ 2.304%)	4,048,774	4,153,217 (+ 2.580%)	4,153,124 (+ 2.577%)	4,056,850	4,113,818 (+ 1.404%)
Bases aligned	596,380,132	596,611,260 (+ 0.039%)	599,244,753	599,601,399 (+ 0.060%)	599,528,267 (+ 0.047%)	553,338,901	548,757,802 (− 0.828%)
Bases unaligned	62,191,423	61,960,355 (− 0.372%)	59,307,655	58,949,429 (− 0.604%)	59,023,102 (− 0.480%)	105,271,191	109,852,201 (+ 4.352%)
Bases unaligned from clipped reads	22,349,569	22,380,472 (+ 0.138%)	27,442,533	27,690,552 (+ 0.904%)	27,575,464 (+ 0.484%)	3,625,336	3,589,573 (− 0.986%)
Bases mismatched	3,458,029	3,308,480 (− 4.325%)	3,596,667	3,458,707 (− 3.836%)	3,455,296 (− 3.931%)	2,164,703	2,029,911 (− 6.227%)
Bases inserted	65,210	65,151 (− 0.090%)	84,358	85,938 (+ 1.874%)	85,397 (+ 1.232%)	26,674	26,763 (+ 0.334%)
Bases deleted	52,272	51,165 (− 2.118%)	70,324	72,082 (+ 2.500%)	70,347 (+ 0.033%)	11,793	11,756 (− 0.317%)
Non-primary alignments	246,092	246,540 (+ 0.182%)	539,309	724,904 (+ 34.414%)	724,613 (+ 34.360%)	969,452	755,436 (− 22.076%)
Time (s)	533	721	10,711	4457	4540	312	517

**Fig. 2:**
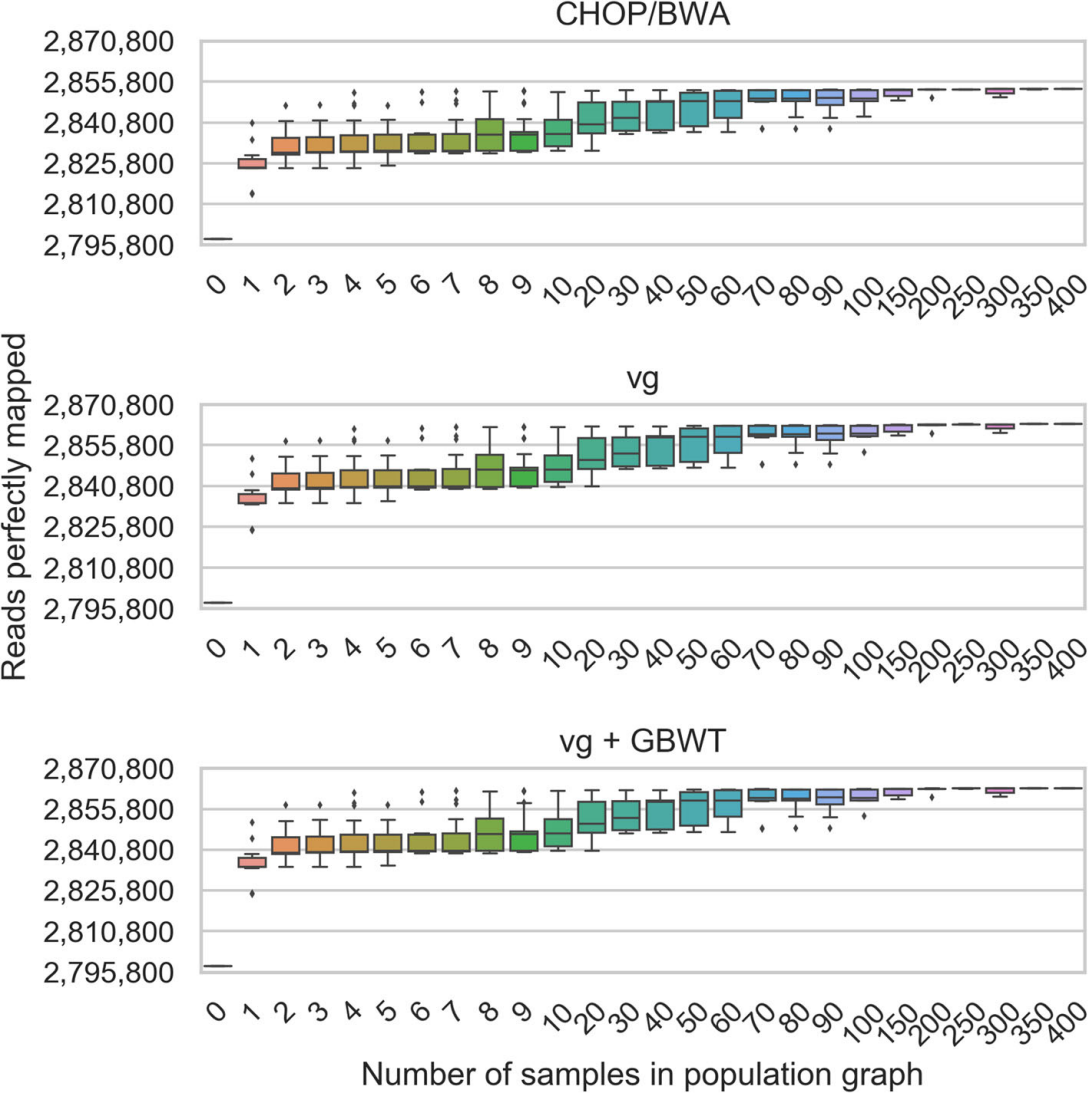
Perfectly aligned read count for SRR833154 alignments to different sized population graphs, containing between 0 (only H37Rv, the linear reference) and 400 samples for both, when using CHOP/BWA and vg with and without haplotyping to align reads to the graph

Using these measurements as a baseline, read to graph alignments were compared between CHOP/BWA and vg. The different graph constructions of CHOP and vg were found to have minimal effect on alignments as shown in Additional file 1: Figures S5 and S6. Figure 2 shows the increase in perfectly aligned reads using both CHOP/BWA and vg as more samples are incorporated into the graph (similar plots for the number of unaligned reads and mismatches can be found in Additional file 1: Figures S7 and S8). Table 1 shows the alignment results for the MTB graph with 400 samples.

Figure 2 shows that incorporating more variation from samples into the population graphs increases the number of aligned bases, which is further demonstrated in Additional file 1: Figures S7 and S8. Spread is a consequence of sampling when building the population graphs, where samples that are closely related to the hold-out sample will yield greater improvement than distantly related samples, further demonstrated by the reduction of spread as sampling size increases.

By comparing vg and vg+GBWT, the effects of haplotyping can be observed, noting a drop in the number of aligned reads. This is to be expected as the index space has been constrained to only the haplotypes.

The baseline alignments to H37Rv already highlighted that the aligners perform differently. However, throughout the course of the experiments, nearly all alignment criteria show the same trend for both CHOP/BWA and vg. The exception being the number of unaligned reads, which steadily decreases with vg, whereas this is not as pronounced when using CHOP/BWA. To better disentangle the aligner-specific differences of CHOP/BWA and vg, we directly compared CHOP and vg+GBWT by aligning to the CHOP null graphs using vg (denoted as CHOP/vg), as described in Additional file 1: Section 7. We found that although we do observe differences in alignments between CHOP/BWA and vg+GBWT, these are merely caused by differences in the aligners. This was confirmed when comparing vg+GBWT with CHOP/vg which has shown nearly identical alignments (Additional file 1: Table S2). Alternatively, the alignment differences between CHOP/BWA and vg+GBWT could be minimized by optimizing the parameters of the aligners, as we used only default settings for both.

In a similar setting, we compared to HiSat2 (Additional file 1: Section 8), results shown in Table 1. While HiSat2 aligns much faster than CHOP/BWA and vg(+GBWT), this can be attributed to its lower sensitivity, having far more unaligned reads in both the baseline and the graph alignments. Surprisingly, the number of unaligned reads increases in the graph alignments with respect to the linear genome, while the number of non-primary alignments decreases. This may indicate that not all sequence in the graph is indexed.

Together, these experiments clearly show that when increasingly more genomes are populating a variation graph, (1) more reads can be aligned (with fewer mismatches), (2) constraining the alignment by haplotype does not adversely affect alignment, and (3) that both haplotype constrained aligners (CHOP and vg+GBWT) perform similarly (as expected).

### CHOP scales to *Homo sapiens*

To further evaluate scalability and sensitivity of CHOP, we used chromosome 6 (170 Mb) of the GRC37 assembly in combination with the 1000 Genomes phase 3 variation data [33]. The constructed graph has 14,744,119 nodes and 19,770,411 edges and encodes a total of 5,023,970 variants (4,800,102 SNPs, 97,923 insertions, and 125,945 deletions). Note that the variation set included diploid phasing of 2504 individuals, which was incorporated into the graph as 5008 paths (2 paths per sample), and additionally 1 path that represents the reference genome. Within the population, most variation (58.42*%*) is shared between at least 2 or more samples (Additional file 1: Figure S9). We used 15 single-end read sets from the 1000 Genomes phase 3 for the graph alignments (Additional file 1: Table S4) that were filtered to include only reads aligned to chromosome 6 or that could not be aligned anywhere on the genome (average read set size of 3,026,069).

CHOP was set to report 100-length paths through the graph to match the read length, which yielded 11,359,686 nodes in *G*^*E*^. The memory usage and time taken for indexing were dominated by CHOP, BWA indexing accounting for only 6.95*%* of indexing time and a fraction of memory required. We attempted indexing with vg and vg+GBWT for paths of up to 104 bp (*k*=13, 3 doubling steps), but this did not finish due to memory constraints (500 GB). Instead, doubling was lowered to 2, and paths up to 52 bp were indexed. By incorporating haplotyping in vg, the indexing requires substantially more time (6 × longer) than indexing without haplotyping, whereas memory usage remains constant. The read sets were aligned to both the linear reference of chromosome 6 and the graph representation, using either CHOP/BWA, vg, or vg+GBWT, which is summarized in Table 2.

**Table 2:** Mean of alignment results from 15 samples from the 1000 Genomes data when aligning to (1) the reference genome sequence of chromosome 6 (column GRC37) and (2) the population graph created from the 5008 haplotypes, for both CHOP/BWA and vg with and without haplotyping

	1000 Genomes samples, read length = 100						
Alignment criteria	BWA	CHOP/BWA	vg	vg	vg + BGWT	GraphAligner	GraphAligner
	GRC37	Graph (*n* = 2504)	GRC37	Graph (*n* = 2504)	Graph (*n* = 2504)	GRC37	Graph (*n* = 2504)
Reads aligned	2,542,399	2,543,522 (+ 0.044%)	2,684,925	2,726,051 (+ 1.532%)	2,717,972 (+ 1.231%)	2,664,609	2,630,670 (− 1.274%)
Reads unaligned	483,670	482,548 (− 0.232%)	341,144	300,018 (− 12.056%)	308,098 (− 9.687%)	361,460	395,399 (+ 9.389%)
Reads perfectly aligned	1,794,564	1,977,952 (+ 10.219%)	1,807,158	1,993,967 (+ 10.337%)	1,993,469 (+ 10.310%)	1,789,327	1,950,435 (+ 9.004%)
Bases aligned	251,122,992	251,516,725 (+ 0.157%)	254,518,323	255,911,471 (+ 0.547%)	255,578,370 (+ 0.416%)	258,684,466	256,773,126 (− 0.739%)
Bases unaligned	51,439,949	51,070,534 (− 0.718%)	48,029,518	46,654,663 (− 2.863%)	46,995,159 (− 2.154%)	41,030,266	43,649,514 (+ 6.383%)
Bases unaligned from clipped reads	1,801,947	1,846,687 (+ 2.483%)	12,716,162	15,699,089 (+ 23.458%)	15,245,035 (+ 19.887%)	203,221	177,659 (− 12.578%)
Bases mismatched	1,270,981	969,087 (− 23.753%)	1,198,917	953,800 (− 20.445%)	940,371 (− 21.565%)	4,681,045	3,931,955 (− 16.003%)
Bases inserted	43,979	19,661 (− 55.296%)	59,078	40,786 (−30.962%)	33,391 (− 43.480%)	2,541,719	1,925,093 (− 24.260%)
Bases deleted	61,659	32,355 (− 47.526%)	73,131	44,555 (− 39.075%)	41,040 (− 43.882%)	464,085	415,508 (− 10.467%)
Time alignment (s)	544	1807	19,996	10,436	10,871	916	2102
Memory alignment (MB)	412	5534	837	3296	4389	3047	14,446
Time indexing (s)	186	CHOP, 43,625; BWA, 3256	37	5751	33,619	NA	NA
Memory indexing (MB)	245	CHOP, 56,969; BWA, 3813	269	45,670	45,868	NA	NA

We observed the same improvement of moving to a graph representation as in MTB, although more extensive, given that more variants, including indels, are incorporated into the graph.

Given the different path lengths used, time cannot directly be compared between CHOP/BWA and vg. Nevertheless, it is unclear why vg took substantially more time to align than CHOP/BWA, especially when aligning to the linear reference. Differences (relative to MTB) between vg and vg+GBWT have become more prominent, given that more samples are incorporated within the graph. Note that vg+GBWT is slower than vg in both indexing and alignment. This is because the GBWT index, used in vg+GBWT, is build and used alongside the GCSA2 and xg indexes that are already present in vg. The gain of the GBWT index is therefore primarily to correct the alignment process by adding the haplotype constraints.

We observed substantial differences between CHOP/BWA, vg, and vg+GBWT with respect to the decrease of unaligned reads − 0.23*%* versus − 12.06*%* and − 9.69*%*, and the increase in read clipping + 2.46*%* versus 23.46*%* and 19.89*%*, respectively. To evaluate this aligner-induced difference, we extracted all reads that aligned exclusively onto the graph, which amounted to 21,661 reads in CHOP/BWA and 616,900 in vg. Additional file 1: Figure S10 displays the distribution of the number of aligned bases for each of those reads. Nearly all (97.61*%*) of the newly aligned reads by vg had a length of between 15 and 30 bases, either induced by clipping or extensive base insertion/deletion. However, from 30 bases and up, the aligners display very similar profiles, with a comparable number of newly aligned reads. At 69 bp, both aligners display a peak, the newly aligned reads corresponding to this peak all align to the same region in the graph. This region closely resembles human mitochondrial DNA, which was excluded from the initial reference alignments. This has led to an increased number of unaligned mitochondrial sequencing reads in the dataset that were aligned to the graph (Additional file 1: Section 11).

By simulating read data of chromosome 6, we measured the accuracy of alignments to graphs and linear references. We observed that by building graphs from subsets of available variants (selected based on allele frequency in the population), alignment performance could be improved (Additional file 1: Section 12). We observed similar improvements when aligning reads to a graph build from the alternate alleles of the MHC region of chromosome 6 (Additional file 1: Section 13).

Additionally, we compared CHOP/BWA to Graphtyper (Additional file 1: Section 14). As the main purpose of Graphtyper is genotyping and variant calling (and thus does not output alignments), we also called variants from the CHOP/BWA alignments. Although Graphtyper did not detect any new variants when aligning reads from sample HG00308 to the 1000G chromosome 6 graph, it did genotype variants (144,800 out of 5M, after filtering). Contrarily, CHOP/BWA did detect 1212 variants from which 57 remained after quality filtering. Note that variant calling the CHOP/BWA output was more than 2 orders of magnitude faster than Graphtyper, while using an order of magnitude less memory.

We failed to index the graph with HiSat2 due to extreme memory usage (200 GB within 709 s) and concluded that it does not scale to population graphs of this complexity (Additional file 1: Section 8). We also compared to the long read aligner GraphAligner (Additional file 1: Section 15), and the results are shown in Table 2. Note that GraphAligner is optimized for long reads and could generate suboptimal results when short reads are utilized. GraphAligner was able to index and align to the 1000G chromosome 6 graph, with alignment times close to CHOP/BWA. The alignment statistics do, however, similar as in the case of HiSat2 for MTB, show a counter-intuitive decrease in the number of aligned reads when aligning to the 1000G graph instead of the linear genome.

**Fig. 3:**
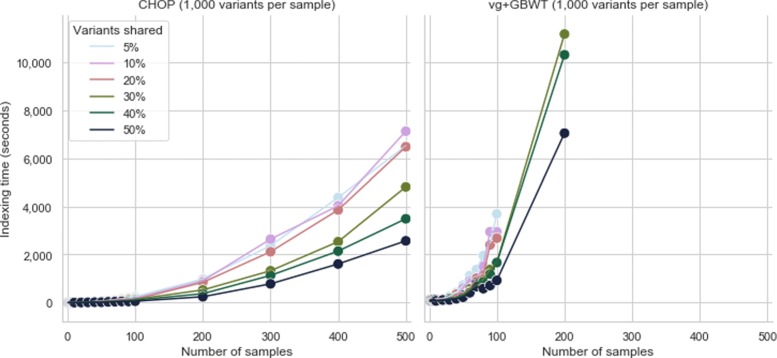
CHOP and vg+GBWT indexing time (s) of graphs with increasingly more encoded samples, where each sample contributes 1000 variants to the graphs. The coloring indicates different probabilities of sharing variants within the simulated population. For instance with a probability of 5%, 95% of all sample variation will be unique to that particular sample, while the remainder is shared with 1 or more other samples. Missing points in the plots indicate that the indexing failed by either exceeding 4 h of compute time or peak memory of 80 GB. More details can be found in Additional file 1: Section 17

To better grasp the practical limitations of CHOP, we indexed the previously introduced graphs for varying *k* values (Additional file 1: Section 16), where we note an approximately linear growth in indexing time and memory usage. Additionally, we further compared CHOP and vg+GBWT using simulated variation graphs with varying degrees of variation, the number of encoded genomes, and shared variation between genomes under set memory and time constraints (Additional file 1: Section 17). Figure 3 highlights the differences in indexing time of CHOP and vg+GBWT for simulated graphs with samples that encode 1000 variants each. We show that CHOP indexes faster and more efficiently than vg+GBWT and could handle more complex graphs (CHOP could index 92.75% of all simulated graphs, whereas vg+GBWT managed to index 79.28%).

**Fig. 4:**
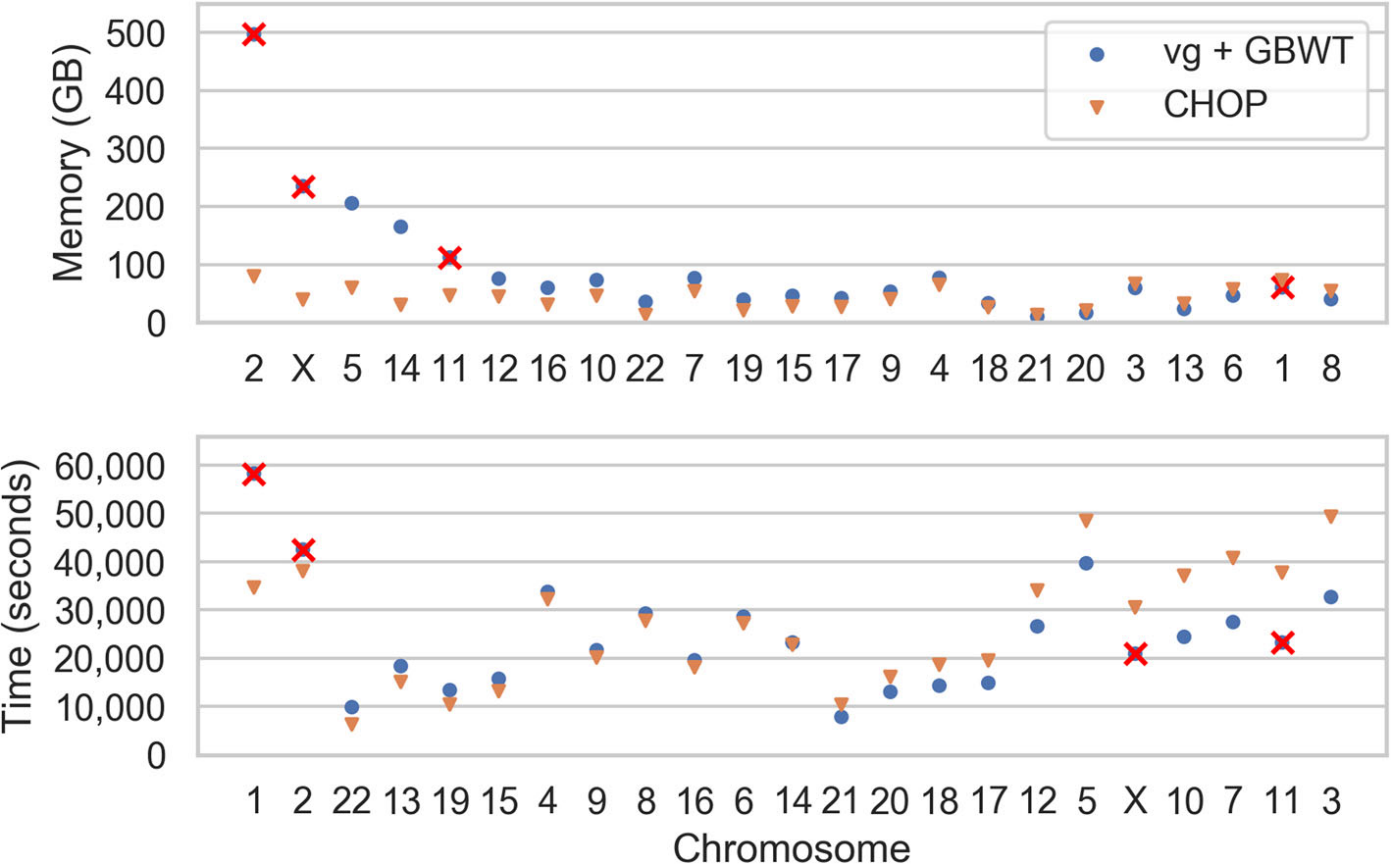
Peak memory footprint and time required for indexing the human chromosomes using CHOP and vg+GBWT. Chromosomes are ordered according to the relative differences between CHOP and vg+GBWT. Chromosomes 1, 2, 11, and X are crossed out for vg given that these ran out of memory constraints (> 500 GB) or disk space constraints (> 6 TB)

Finally, we performed alignments to the full human genome. We constructed graphs of each chromosome encoded with the variants as reported by the 1000 Genomes Project phase 3. Cumulatively, these graphs have 248,677,280 nodes and 33,3561,973 edges and encode a total of 84,745,123 variants (81,382,582 SNPs and 3,362,541 indels). We indexed the graphs with both CHOP for 100-length paths and with vg+GBWT for 52-length paths; the peak memory usage and time required for indexing are reported in Fig. 4. Note that chromosomes 1, 2, 11, and X could not be indexed with vg+GBWT, due to the graph complexity (at times more than 50 variants in a 50-bp window) leading to excessive memory usage (> 500 GB) or disk usage (> 6 TB), more details in Additional file 1: Section 18. To be able to handle these chromosomes, the graphs would have to be simplified prior to indexing. Indexing with CHOP yielded 103,509,254 nodes in *G*^*E*^, which increased the total sequence space by 14 ×. We again used BWA and aligned the sample ERR052836 to both the linear reference genome and the graph, where we noted a 2–3 × (13,704 to 37,826 s) increase in read alignment time to the graph with respect to the linear genome.

## Variation integration

**Fig. 5:**
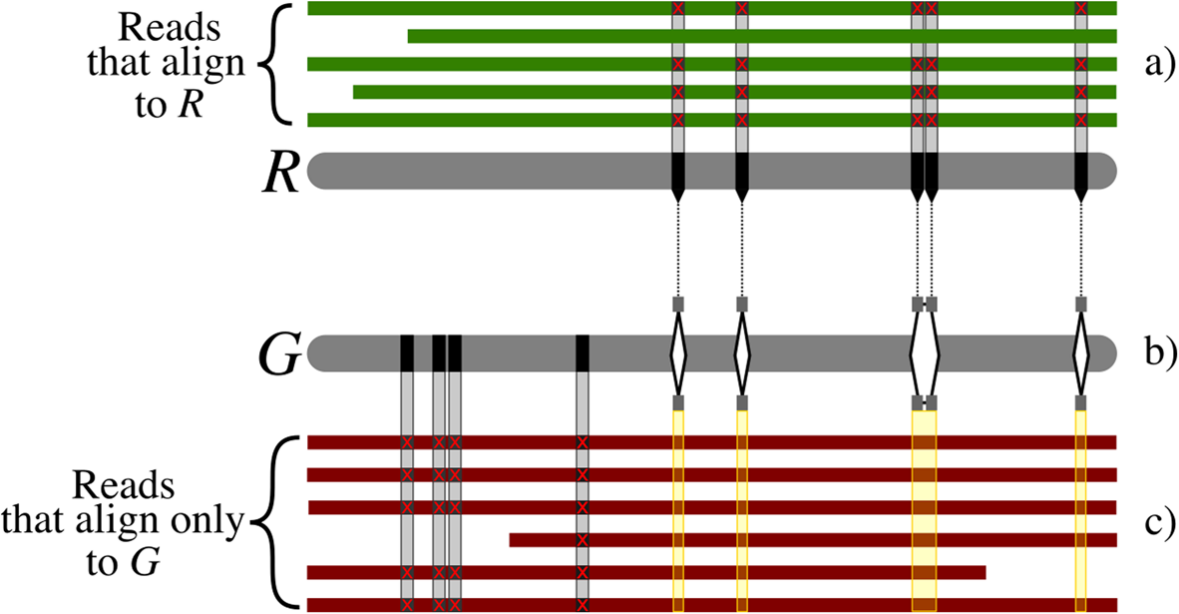
**a** Schematic alignment of SRR833154 reads to the reference *R*, H37Rv, with subsequent variant calling detecting five high-quality SNPs in this particular region. **b** These and all other SNPs across the genome are integrated with the reference into graph *G*, followed by the alignment of the same reads. **c** Reads that previously did not align to *R*, now align onto a haplotype of the graph *G*. Formation of a pileup allows for the detection of four new variants in this region

As graphs span a larger search space, we investigated how this affects read alignment and variant calling. Theoretically, encoding more distinct sequences in a graph should enable alignment of more reads and potentially allow new variants to be called. To evaluate this, variants were integrated using a feedback loop. First SRR833154 reads were aligned to H37Rv using BWA, then variants were called using Pilon. Variants were quality filtered down to 838 SNPs and then used to construct a graph with H37Rv (now thus including two genomes). The same set of reads was then aligned onto the graph, and variants were called. We expected that the additional context offered by the graph would point to previously undiscovered variants. An example of this is schematically shown in Fig. 5a; in Additional file 1: Figure S19, we show an example of such newly aligned reads.

Integrating variants in a graph (Fig. 5b) and realigning reads to the graph allowed reads to follow a path within the graph that best matches. This in turn allowed for reads that previously were not able to align now to be aligned (Fig. 5c). As a result, 19 (+ 2,26*%*) new high-quality variants could be called from these new aligned reads.

## Discussion

Population reference graphs have the potential to improve sequencing analyses by taking into account within-species genetic diversity during the process of aligning sequencing reads. This can potentially improve various downstream analyses, like variant calling.

A challenge for aligning reads to a graph efficiently is to find exact matching seeds of a fixed length *k* that can span the edges of the graph. Searching through an enumeration of all possible *k*-length paths in the graph is computationally challenging as the exponential growth of paths adversely affects the memory footprint as well as the alignment time. This puts practical limits on the variation that can be encoded in the population graph. We suggest the use of haplotype information to constrain this exponential growth. Doing so, the genetic linkage between neighboring variants can be exploited to counter, not only the computational problems, but also the number of false-positive matches that arise due to unobserved combinations of variants (variants encoded on different alleles). Recently, [37] has proposed an alternative approach to circumvent the computational challenge of exponential path growth in graphs by combining a graph index with read chunk indexes, exploiting the limited *k*-mer space of reads relative to that of the graph. It will certainly be interesting to see how a haplotyping approach can be combined with this method.

Here, we introduced CHOP, a method which converts a haplotype-annotated population graph into a set of sequences that covers all observed *k*-paths. It does this by transforming the population graph into a null graph (a graph devoid of edges) such that every observed *k*-path is represented in 1 of the resulting unconnected nodes. As the resulting set of sequences (the null graph) is a compressed representation of all *k*-paths through the graph, it becomes feasible to use values for *k* that are equal to the length of typical NGS reads (e.g., 100 to 150). For this reason, an additional advantage of CHOP is that any NGS read aligner (e.g., BWA or Bowtie) can be used to align reads onto the created null graph. As every position in the null graph can be translated back to a position in the initial population graph, we can effectively perform scalable read to graph alignment. The advantage of CHOP over existing graph-based alignment approaches is that we propose a solution to incorporate the haplotype constraint throughout the whole procedure and thus truly do not suffer from a combinatorial explosion of possible paths as our complexity is bounded by the number of haplotypes encoded in the graph. Through this solution, CHOP does not have to rely on filtering or pruning the graph in any way to scale to complex population graphs.

With CHOP, we followed an approach more closely related to string/overlap graphs, instead of a de Bruijn graph (DBG) approach, as they can better handle cycles induced by repetitive sequence. There is a key difference with a (compacted) DBG approach as a DBG is always constructed for a fixed value of *k*. To keep the index manageable, this *k* value needs to be relatively small. However, for a small value of *k* (commonly ∼15 is being used), the resulting DBG will contain cycles that are introduced by repeated *k*-mers. These cycles prevent parts of the genome/graph to be addressed uniquely. This, while the input data structure (the variation graph), has no ambiguity whatsoever. Representing the variation graph as a DBG, therefore, inevitably causes a loss of information. To further clarify this difference, we can state that with CHOP, every position in the variation graph maps to at least one unique position in the index (null graph), while with a DBG approach, multiple positions in the variation graph can map to the same position in the DBG, also exemplified in Additional file 1: Figure S20. The size argument for DBGs follows from the fact that these repeated *k*-mers are stored only once, which is exactly what introduces the ambiguity in the first place. Therefore, the higher the compression rate, e.g., using bloom filters, the more ambiguity is introduced in the representation. Bloom filters are probabilistic data structures that balance the need to store these very large hash tables against the integrity of the resulting representation (as they allow for colliding hash functions, e.g., edges in the DBG). Although these representations are a computational answer to the need to store and query very large hash tables (e.g., DBGs with “large” *k* values), they actually further impair the representation of the underlying variation graph by allowing for non-existent edges.

We have shown that read alignment using CHOP in combination with BWA (CHOP/BWA) easily scales to the whole human genome, encompassing all 84,745,123 variations reported by the 1000 Genomes Project (2504 individuals). The memory footprint of CHOP per human chromosome is less than 80 GB and takes under 50,000 s.

Furthermore, we have shown that graph indexing and alignment with CHOP/BWA resulted in more aligned bases compared to aligning to the linear reference genome. Also, we found that the number of aligned bases grows proportionally with the number of incorporated variants (samples). Interestingly, the amount of sequence required to store the resulting compressed *k*-paths grew faster than the time needed to perform the alignments. We attribute this to an increase in the number of exact matching reads, which decreases the need for extending initial seeds during the alignment, which is a computationally demanding task.

We extensively compared CHOP/BWA to vg, which is the current state-of-the-art toolkit for working with population graphs and includes a read alignment module. Vg uses GCSA2 indexing, an extension of the BWT for population graphs, supporting exact query lengths of up to 256 bp. Recently, vg has been expanded to facilitate haplotype-constrained alignment using the GBWT index, a graph extension of the positional Burrows-Wheeler transform. Note however that vg still requires the construction of the GCSA2 index together with the GBWT to perform haplotype-constrained alignment, which still risks the exponential growth of paths while indexing, an issue that does not occur with CHOP.

When comparing read alignments of CHOP/BWA with vg and vg with haplotyping (vg+GBWT) on population graphs of both *Mycobacterium tuberculosis* (MTB) and humans, we found very similar alignment results, as expected. However, compared to CHOP/BWA, alignment took five to six times longer with vg(+GBWT). Furthermore, CHOP scaled better with complex graphs, which we have shown by indexing and aligning to a graph of the full human genome. Although vg and vg+GBWT were able to index most of the chromosomes, this was only when we adapted path lengths of *k*=52, which is approximately half the length of CHOP *k*-paths. Then, still for a few complex chromosomes, indexing failed using vg. Moreover, we have shown CHOP’s scalability up to *k* = 300 for this particular graph (Additional file 1: Figure S14).

Our comparisons with HiSat2 and GraphAligner has shown that HiSat2 does not scale to the human variation graph encoding all 1000G variants, whereas GraphAligner does. We should report here that HiSat2 has been shown [38] to scale to human by first making a preselection of the variants that are included in the graph, which also reduces the number of false-positive alignments. CHOP eliminates the need for prefiltering variants leaving more freedom for users to make this decision or, otherwise, drastically increasing the number of genomes with high-fidelity variants in the variation graph. Moreover, for both HiSat2 and GraphAligner (possibly attributable to its optimization for long reads), the alignment results are not in agreement with those of vg(+GBWT) and CHOP/BWA.

Interestingly, the read alignment results did not differ much between a haplotype-constrained aligner and a non-haplotype-constrained aligner. This can be best observed when comparing vg with vg+GBWT, because they utilize the same aligner and parameters. Although, the number of aligned reads increases by 1.5*%* when considering all *k*-paths (vg) in the human population graph with respect to the linear reference genome, as opposed to an increase of 1.2*%* when considering haplotype-constrained *k*-paths (vg+GBWT). Inspection of the additionally aligned reads seems to indicate that most of these alignments are the result of spurious matches induced by unsupported sequence combinations. Altogether, this seems to suggest that indexing all possible *k*-paths does not add much value while at the same time increasing the chance of false-positive alignments. Note that non-haplotype-constrained alignment might still be useful when one expects that the genome to be aligned is more distant to the encoded genomes in the variation graphs, and consequently, recombined haplotypes could guide the alignment.

The advantage of limiting *k*-paths to observed haplotypes is further supported by our observation that population graph alignment improves with respect to a linear reference genome when not all observed variation is incorporated into the graph (Additional file 1: Section 12). Our simulations on a 1000G sample has shown that improved read alignments (as identified by a reduced number of false-positive/negative alignments) can be achieved when the allele frequency of a variant is considered when building the population graph. Simply put, if the frequency of a variant increases, it is more beneficial for read alignment to incorporate such variants in the population graph, at a minimal cost of introducing false positives. Note that rare variants within a sample can still be called after the reads are mapped to the graph; they are just not used when building the population graph.

Graphs that serve as input to CHOP should encode phased variant calls. While this information is not typically encoded in variant call formats, it is required at only short ranges (related to the value for *k*) and should be readily be available from typical sequencing experiments. In our experiments, the complexity of incorporated variation was limited to SNPs and small indels. Therefore, the benefit of a population graph on increasing the number of aligned reads was limited, since the identification of SNPs and small indels are well identifiable using a linear reference genome. However, CHOP is not restricted to graphs constructed from variant calls but can handle any acyclic sequence graph, e.g., as generated from multi-whole-genome alignments or haplotype-aware de novo assembly algorithms [39, 40] (Additional file 1: Section 13). Consequently, both short (SNPs/indels) and long range (structural variants) haplotypes can be incorporated in the graph and in the resulting index. Incorporating larger structural variations will lead to more substantial improvements. One should realize, however, that incorporating structural variation increases the amount of repeated sequence in the graph, e.g., the incorporation of mobile element insertions and repeat expansions, which will lead to an increase in ambiguously aligned reads.

CHOP does not directly support long reads or paired reads. Long reads, with *k* typically exceeding > 10 kbp, will still lead to an intractable number of haplotype-constrained *k*-paths. However, the alignment of long reads generally depends on the detection of short seeds in the first place, which can easily be extracted from the compressed representation of *k*-paths generated by CHOP. Hence, long read alignments may be seeded, where a subgraph can be extracted (based on the seeds) and aligned to with partial order alignment. Alternatively, the heaviest weighted sub-path can be extracted from the graph [41], followed by a typical sparse alignment on that linear sequence. For paired reads, reads are aligned to discrete *k*-paths, where an aligner such as BWA cannot directly measure the distance between any distinct *k*-path. Note that read pairing should be possible based on the haplotyped paths in the graph, namely, that the distance between any two nodes in the graph will follow a distribution of distances (of each reachable haplotype) and that this enables the evaluation of read pairs during read alignment (in a stand-alone aligner) or as a post-processing step.

In a comparison with Graphtyper, we have shown that by using CHOP/BWA, we were able to detect new variants when aligning reads to the 1000G variation graph, whereas Graphtyper is able to genotype variants in a large population. Finally, we have shown that by iteratively integrating aligned sequencing reads derived from one genome to the linear reference genome using the graph representation improves variant calling. Aligning additional reads led to the additional calling of variants, which subsequently could be merged with the built population graph, reiterating the whole process (multiple times). This application of population graphs is similar to iterative realignment methods, such as ReviSeq [42], but is solved in a more general way when using population graphs as the starting point.

## Methods

### Population graph definition

Population graphs were constructed given existing reference genomes and sets of variations, called from linear reference alignments (Additional file 1: Section 5). Nodes within graphs are labeled, encoding genomic sequences that may be shared within multiple haplotypes, which are in turn connected by directed edges. Traversing a sequence of edges, i.e., a path, will describe either a mixture of haplotypes or an observed haplotype within the graph.

### Population graph specification

A population graph *G*=(*V*,*E*) is defined as a set of nodes *V*={*v*_0_,…,*v*_*N*_}, where *N*=|*V*|, and a set of edges *E*. Each of these edges is an ordered pair of nodes (*u*,*v*)∈*E*, where node *u*∈*V* is connected to node *v*∈*V*. As *G* is a directed graph, it holds that for any edge (*u*,*v*)∈*E*, (*u*,*v*)≠(*v*,*u*).

For each node *v*∈*V*, the in-degree, in(*v*), is defined as the number of incoming edges to that node, i.e., the number of distinct edges (*u*,*v*)∈*E* for any *u*∈*V*. Conversely, the out-degree of node, *v*, out(*v*), is defined as the number of outgoing edges from that node.

Every node, *v*, is assigned a sequence of characters, *S*, consisting of the alphabet *Σ*={*A*,*T*,*C*,*G*}, such that *v*_*S*_=*S*[0, *n*−1], wherein *S*[*i*]∈*Σ* for all *i*, and the length of the sequence, *n*, is defined as *n*=|*v*_*S*_|. The range of any such sequence for any node, *v*∈*V*, lies between 1≤|*v*_*S*_|≤*L*, where *L* is the length of the largest recorded sequence. Any substring of a sequence, *S*, is denoted as *S*[*i*, *j*]. Two types of substrings in particular are prefixes *S*[0, *j*] and suffixes *S*[*i*, *n*−1], which describe the left and right flanks of any sequence *S*, respectively.

A path, *P*, where *P*=*u*_0_⋯*u*_*q*−1_, is any consecutive series of nodes (*u*_*i*_, *u*_*i*+1_)∈*E* for all *i*<*q*, where *q*=|*P*| is the total number of nodes on the path. If a path exists between any pair of nodes in a graph, it is a connected graph, i.e., there are no unreachable nodes. The sequence, *S*, of a path, *P*_*S*_, is the concatenation of sequences contained in the nodes, such that *P*_*S*_=*u*_0*S*_⋯*u*_(*q*−1)*S*_.

Given haplotyping information, the graph *G* is augmented with a set of haplotypes, *H*, where *H*={*H*_0_,…,*H*_*h*−1_}, where *h*=|*H*| is the number of observed haplotypes. Every edge (*u*,*v*) is assigned a subset of *H* denoted as (*u*,*v*)_*H*_, which describes the haplotypes that pass through the edge. Each encoded haplotype is represented by a path traversal through *G* and may overlap other haplotypes.

Let *G*^*E*^ denote the null graph of *G* such that *G*^*E*^=(*V*^′^,*∅*), where *V*^′^ originates from merging nodes in *V* (details of which are to follow in the subsequent section).

### Constructing the null graph

**Fig. 6:**
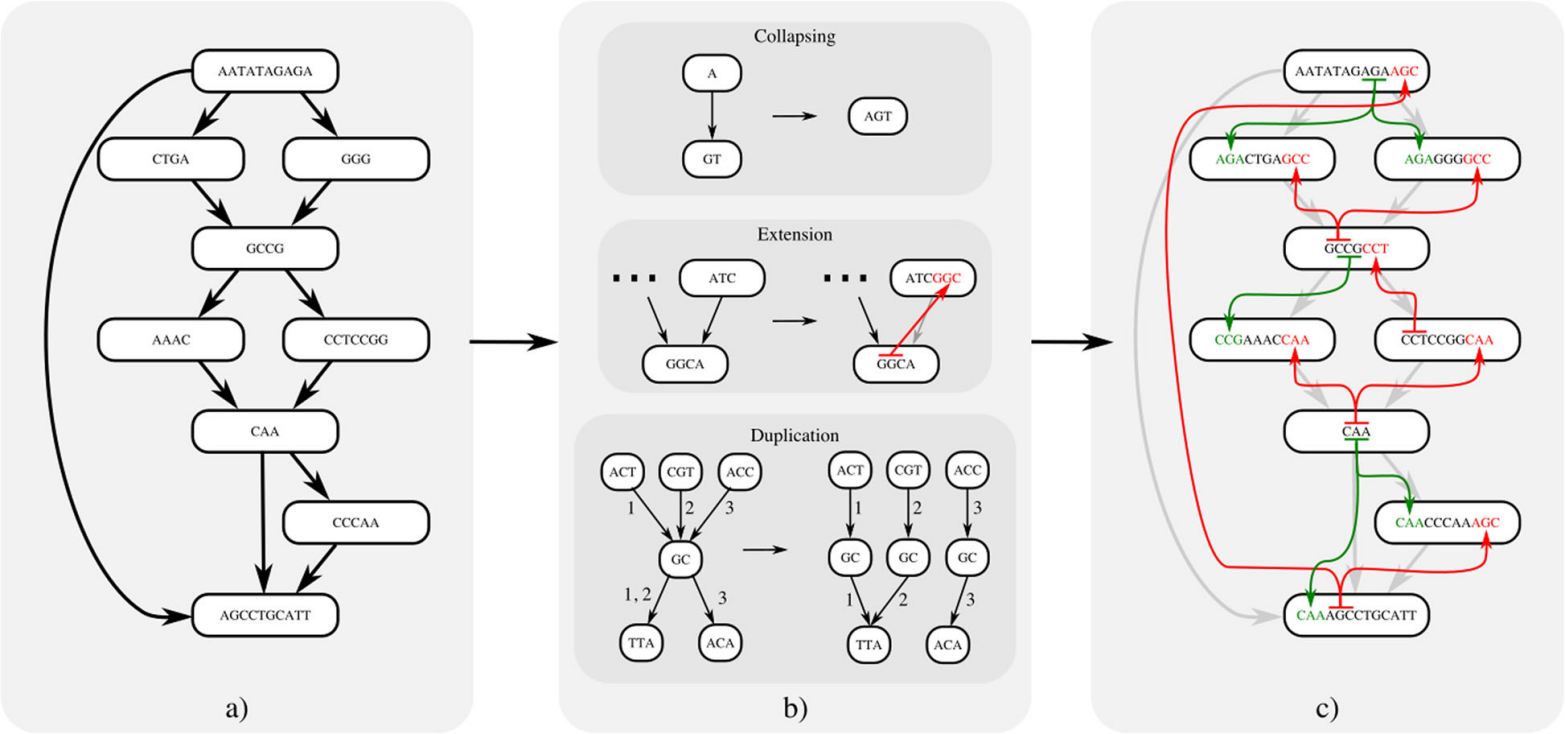
Reporting the haplotyped *k*-paths in the population graph *G* transforms it into the null graph *G*^*E*^, here *k*=4. **a** A population graph with sequence encodings on the nodes. **b** Indexing of *k*-paths based on three operations; Collapsing, merging adjacent nodes. Extension, assigning *k*-length substrings as prefixes or suffixes between adjacent nodes. Duplication, copying of nodes and redistribution of edges among copies. **c** The null graph encodes all 4-length paths in the original graph, coloring of lines and text denote the origin of assigned prefixes (green) and suffixes (red) (note that colored lines are not edges in the graph)

The purpose of indexing a population graph is to allow for efficient substring queries on the paths that span across nodes and edges of the graph (Fig. 6). Given any non-trivial sized graph, enumerating all possible paths is often unfeasible, given the exponential nature of traversing all combinations of nodes and edges.

CHOP constrains queries through a graph to be part of a haplotype with which the population graph was built. Hereto, CHOP transforms graph *G* into a null graph *G*^*E*^ such that every node in *G*^*E*^ represents a sequence of length *k* or longer and that every substring of length *k* originating from the encoded haplotypes in *G* is also a substring in a node of *G*^*E*^. Meaning that if sequencing reads are true error-free samplings of an underlying haplotype and are of the same length (or shorter) than the chosen value of *k*, they should correspond to a substring of a node in *G*^*E*^. This, in turn, enables the application of any existing read aligner to place reads onto *G*^*E*^. Through this transformation of *G* to *G*^*E*^, all haplotyped paths of at least length *k* in the graph are accounted for. The transformation is driven by three operators: collapse, extend, and duplicate (pseudocode is given in Additional file 1: Listing S1), explained throughout the rest of this section. While the output of CHOP can depend on the order of these three operations, we observed no significant difference in runtime or indexing outcome for different orderings.

### Collapse

The first operation to transform *G* to *G*^*E*^ is collapse, which merges redundant traversals of nodes in the graph. If an edge (*u*, *v*)∈*E* conforms to out(*u*)=1 and in(*v*)=1, then any path that traverses *u* will immediately be followed by *v*. Therefore, it can be considered a redundant traversal such that the sequence on *u* and *v* can be merged without affecting the number of sequences that can be spelled by the graph. To do so, sequence and the corresponding intervals of *u* and *v* are merged after which the outgoing edges of *v* are transferred to *u*, followed by the removal of *v* and the edge (*u*, *v*). We denote this operation as collapsing, defined as *u*||*v* for any edge (*u*, *v*) (as shown in Fig. 6b, pseudocode is given in Additional file 1: Listing S2). The direction of collapsing is guided by minimizing the number of edge reassignments, such that when in(*u*)>out(*v*), *v* is collapsed into *u*, joining the sequence *u*_*S*_=*u*_*S*_⋯*v*_*S*_. Alternatively, *u* is collapsed into *v*, joining the sequence *v*_*S*_=*u*_*S*_⋯*v*_*S*_.

### Extend

**Fig. 7:**
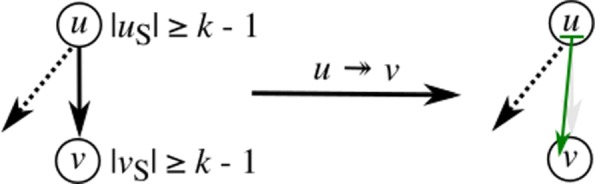
A pair of nodes *u* and *v* where |*u*_*S*_|≥*k*−1 and |*v*_*S*_|≥*k*−1. Note that extension is only possible by prefixing *v* with the right-hand flanking substring of *u*, given that out(*u*)>1. The extension operation denoted as $u \twoheadrightarrow v$ is defined as *v*_*S*_=*u*_*S*_[|*u*_*S*_|−*k*−1,|*u*_*S*_|]⋯*v*_*S*_

**Fig. 8:**
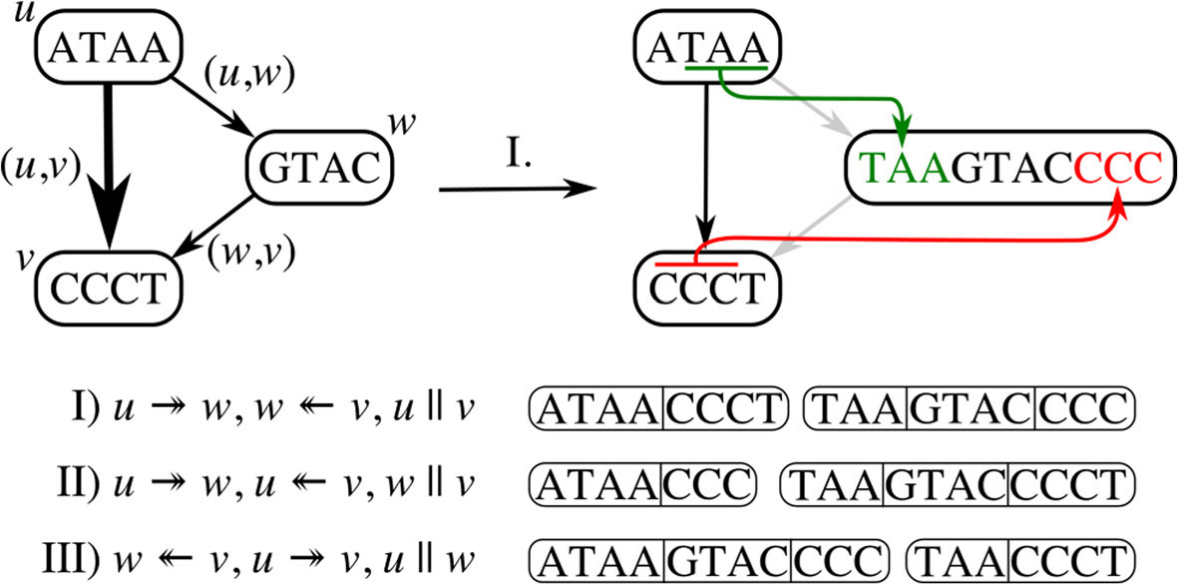
Subgraph in which substring extension for *k*=4 between (*u*,*v*) is not allowed unless either (*u*,*w*) or (*w*,*v*) is resolved first. Three different solutions can resolve this subgraph, and each solution is equivalent in *k*-path space

After collapsing redundant edges in the graph, a number of the remaining edges can be addressed with the extend operation. Extend is based on the observation that all *k*-length substrings that span a single edge (*u*,*v*), i.e., substrings that are defined by substrings of both the sequences of nodes *u* and *v*, can be accounted for by joining a *k*−1-length substring from one node and assigning it to the other. This extension of substrings may happen bi-directionally, namely the *k*−1-length right-hand flank of *u* is extended as a prefix of *v*, denoted as $u \twoheadrightarrow v$, provided that in(*v*)=1 and |*u*_*S*_|≥*k*−1, or vice versa, extending the *k*−1 length left-hand flank of *v* as a suffix of *u*, denoted as $u \twoheadleftarrow v$, provided that out(*u*)=1 and |*v*_*S*_|≥*k*−1 (pseudocode is given in Additional file 1: Listing S3). To illustrate this operation, consider the subgraph in Fig. 7. Within this graph, both nodes *u* and *v* encode sufficient sequence to allow for extension between the two and report a *k*-length overlap, resolving the edge (*u*,*v*). In Fig. 8, a subgraph is shown in which extension is only possible for a subset of edges: (*u*,*w*) and (*w*,*v*). This does not apply for (*u*,*v*), as out(*u*)>1 and in(*v*)>1. This shows a particular situation where only after resolving nearby edges, the subgraph can be sufficiently simplified to resolve all edges. Namely, (*u*,*w*) and (*w*,*v*) must first be resolved before (*u*,*v*) can be solved by a collapse operation. Although the order in which substrings are extended may result in different null graphs, any of these will cover the same *k*-length substrings.

Since extension always concerns a *k*−1-length prefix or suffix, any substring of length *k* that is sampled from the underlying haplotypes will exclusively correspond to either the sequence in *u* or the prefixed sequence in *v* (or vice versa). In other words, by extending and subsequently removing edges in *G*, we introduce overlapping sequence as if we were converting *G* to the repeat-resolved string graph representation of a joint assembly of all genomes in *G* from all possible reads of length *k* [43].

### Duplicate

At times, neither collapse nor extend can be applied to any of the remaining edges in the graph without introducing path ambiguity, a situation in which there are multiple possible candidates to collapse or extend to/from, and choosing any candidate will block off paths to the remaining candidates. In these situations, graph topology must be simplified through the third operation, duplicate. The duplicate operation duplicates a node such that the set of incoming and outgoing edges are split between the duplicated nodes (pseudocode is given in Additional file 1: Listing S4). Duplication allows consequent collapsing, which in turn enables substring extension, such that after a sufficient number of iterations, all edges in *G* can be resolved, either by means of extension or collapsing.

As opposed to methods that aim to track all possible paths through the graph, we suggest the use of haplotype information that is modeled on the edges to constrain the number of necessary node duplications from in(*u*)×out(*u*) to *δ*. Where *δ* is the number of paired incoming and outgoing edges for *u* that have at least one intersecting haplotype. Note that *δ* is bounded by the number of haplotypes encoded in *G* and that there will never be more duplications than haplotypes in any one region of the graph.

**Fig. 9:**
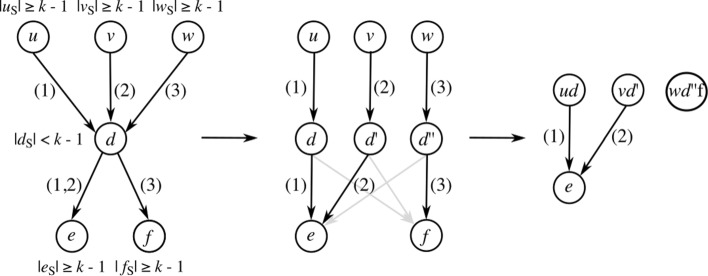
Subgraph with haplotypes: {1,2,3}. Node *d* must be duplicated, as no more edges can be removed through extension or collapsing without introducing ambiguity. By grouping incoming and outgoing haplotypes on *d*, the number of duplications can be reduced. In the resulting graph, edges (*u*,*d*), (*v*,*d*^′^), and (*w*,*d*^″^) can be collapsed. Finally, an extension can be applied to edges (*ud*,*e*) and (*vd*^′^,*e*) which would lead to the null graph. Note that the introduction of grayed out edges is prevented using haplotyping; hence, the edge count is reduced from 6 to 3

To illustrate the idea, Fig. 9 shows a subgraph with haplotypes encoded on the edges. From the haplotyping, we can derive that not all paths through this graph are supported by the underlying haplotypes. For example, the path *u*→*d*→*f* combines sequence segments that are unsupported (the haplotypes between (*u*,*d*) and (*d*,*f*) do not overlap). By excluding these unsupported paths through the graph, the number of duplications for node *d* can be constrained from 6 to 3. This way, the search space for subsequent *k*-length substrings is greatly reduced with respect to reporting all possible paths. Additional file 1: Figure S1 gives the full details about the transformation from Fig. 1a to Fig. 1b.

### Mapping reads to CHOP’s null graph

Established alignment tools can now be used to directly align reads to the null graph representation as long as reads are shorter or equal to *k*+1. Because the sequence modeled on the nodes in *G*^*E*^ is now a composition of sequence originating from adjacent nodes in *G*, the intervals that gave rise to these compositions need to be traced in order to convert the alignment of a read to a node in *G*^*E*^ to a path in *G*. For this reason, during the transformation from *G* to *G*^*E*^, the originating node in *G* and corresponding offset for each prefixed, suffixed, or concatenated sequence is stored alongside the actual sequence. Note that in theory, the defined operations can also be expressed purely in terms of interval operations, excluding any sequence. Given the intervals, a mapping between *G*^*E*^ and *G* is maintained, such that any node in *G*^*E*^ can be traced back to the corresponding path of nodes in *G*. As a result, any alignment to a node in *G*^*E*^ can also be traced to a sub-path of this path, effectively enabling the alignment of reads to graph *G* by using *G*^*E*^ as a proxy (Fig. 1c).

## Supplementary information

**Additional file 1** Additional information. Contains Notes S1-S21, Figures S1–S20, Tables S1–S6, and Listings S1–S4.

**Additional file 2** Review history.
