# Supplementary material for: CHOP: haplotype-aware path indexing in population graphs
Source: Genome Biol. 2020 Mar 11;21:65. doi: 10.1186/s13059-020-01963-y (PMC7066762; doi:10.1186/s13059-020-01963-y)
Supplement: Supplementary file 1 — Additional file 1 Additional information. Contains Notes S1-S21, Figures S1–S20, Tables S1–S6, and Listings S1–S4. [file 13059_2020_1963_MOESM1_ESM.docx]

Additional information

CHOP: Haplotype-aware path indexing in population graphs

Contents

List of Figures 2

List of Tables 2

List of Algorithms 2

1 Transformation to a null graph 3

2 *Mycobacterium tuberculosis* read sets 4

3 Variation growth in *Mycobacterium tuberculosis* population graphs 4

4 Alignment criteria used for evaluation 5

5 Constructing a population graph from known variants 6

6 *Mycobacterium tuberculosis* read alignment to H37Rv and graph 8

7 Aligning to CHOP null graphs with vg 10

8 Comparing CHOP/BWA to HiSat2 11

9 Variation linkage of 1000 Genomes in chromosome 6 12

10 Filtered 1000 Genomes read sets 13

11 Reads aligning to mitochondrial DNA 14

12 Alignment accuracy in chromosome 6 16

13 Aligning to a graph encoding the MHC 17

14 Variation detection with CHOP/BWA and Graphtyper 18

15 Comparing CHOP/BWA to GraphAligner 20

16 Effects of varying *k* size in CHOP 21

17 Simulated graph indexing 22

18 Variant density in human chromosomes 26

19 Variant integration 28

20 CHOP, String graphs, and de Bruijn graphs 29

21 Pseudocode CHOP procedures 30

List of Figures

S1 . . . 3
S2 . . . 4
S3 . . . 6
S4 . . . 6
S5 . . . 7
S6 . . . 7
S7 . . . 8
S8 . . . 9
S9 . . . 12
S10 . . . 14
S11 . . . 15
S12 . . . 16
S13 . . . 21
S14 . . . 22
S15 . . . 23
S16 . . . 24
S17 . . . 25
S18 . . . 27
S19 . . . 28
S20 . . . 29

List of Tables

S1 . . . 4
S2 . . . 10
S3 . . . 12
S4 . . . 13
S5 . . . 17
S6 . . . 20

List of Algorithms

S1 . . . 30
S2 . . . 30
S3 . . . 31
S4 . . . 31

1 Transformation to a null graph

CHOP can through consecutive steps of extension, collapsing, and duplication (as described in the methods) transform population graphs into null graphs. In this edgeless graph representation each node now describes a k-length path through the original graph. In Fig. S1, we describe how the graph in Fig. 1a is transformed into the null graph of Fig. 1b.

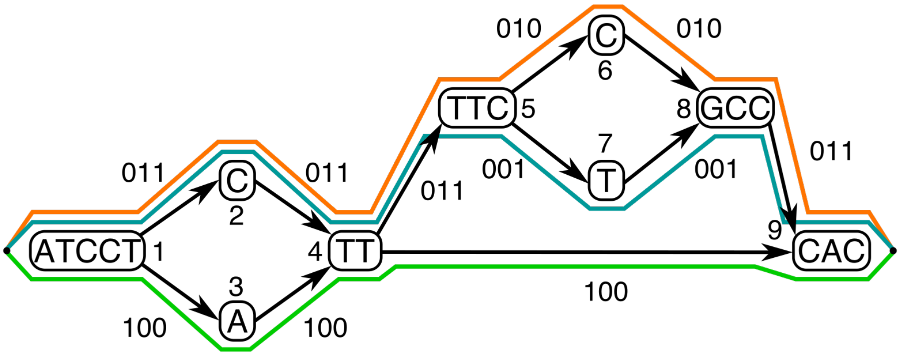

CHOP sequence:

1 ↠ 2
5 ↠ 6
6 ↞ 8
8 ↞ 9
1 || 3, Del(1)
5 || 7, Del(5), Add(4, 7)
Dup(4), Add(4'), Add(3, 4'), Del(3, 4), Add(4', 9), Del(4, 9)
4 || 2, Del(4), Add(2, 7)
4' || 3, Del(4'), Add(3, 9)
2 || 7, Del(2)
9 || 3, Del(9)
8 || 7, Del(8)

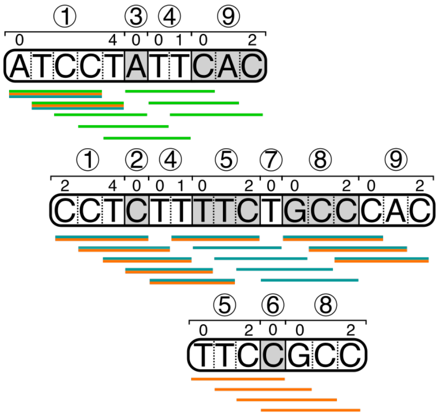

Fig. S1: The same graph as in Fig. 1a, now shown with haplotypes on the edges encoded as bitvectors. Using CHOP, the input graph can be transformed into a null graph. Each of the steps performed by CHOP are shown in sequential order; Extension: $x \twoheadrightarrow y$ (y is prefixed by x), and $x \twoheadleftarrow y$ (x is suffixed by y). Collapsing: $x||y$ (x and y are collapsed into a single node). Duplication: Dup(x), (node x is duplicated).

2 *Mycobacterium tuberculosis* read sets

We obtained the 10 holdout samples from EBI-ENA, as shown in Table S1. All reads have a length of 101 bp, and are single-end.

Table S1: Samples used in MTB experiments, associated read sets are included, with KRITH1/2 accession numbers.

Sample	Read set	KRITH1/2 ID	Read count
TKK-01-0053	SRR833154	G28639	5,263,942
TKK-04-0029	SRR1019154	G47382	5,481,779
TKK-02-0022	SRR1011463	G47310	4,301,550
TKK-01-0093	SRR958234	G38246	9,249,605
TKK-02-0066	SRR924236	G32253	5,168,899
TKK-01-0016	SRR832997	G27617	8,615,425
TKK-02-0051	SRR847783	G32041	7,571,230
TKK-01-0039	SRR833147	G27616	6,443,482
TKK-01-0047	SRR832984	G27644	5,532,779
TKK-01-0033	SRR833024	G27582	7,582,870

3 Variation growth in *Mycobacterium tuberculosis* population graphs

When constructing graphs for the hold-out experiment, progressively more samples (from 1 to 400) are included in the constructed graphs. By including more samples, more variants are incorporated into the graphs, as can be seen in Fig. S2.

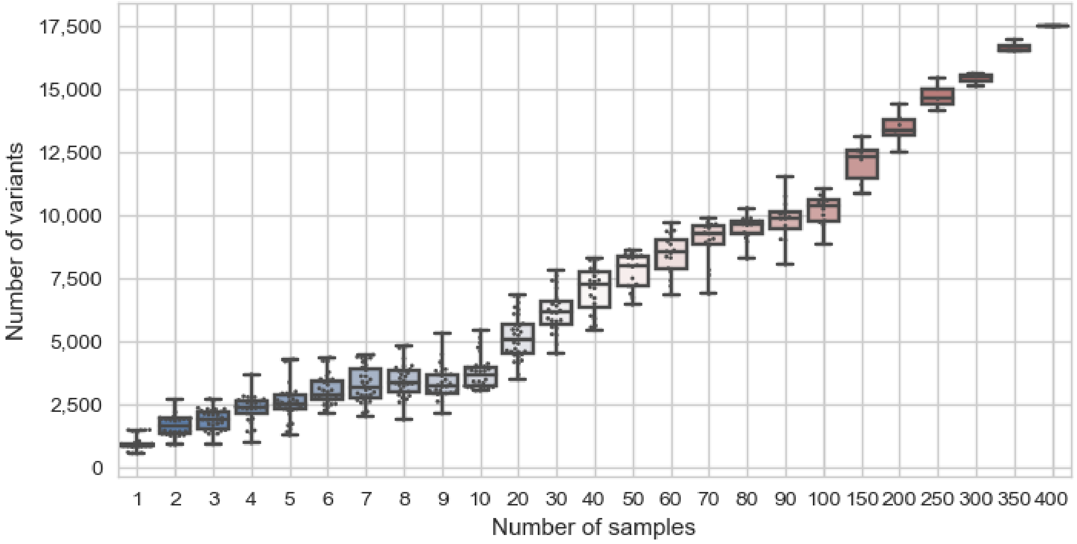

Fig. S2: Variable size variant sampling for VCF-based graph construction.

4 Alignment criteria used for evaluation

To evaluate behavior of different aligners we measure the following criteria: number of mismatches, insertions, deletions, clipped bases, aligned reads/bases, unaligned reads/bases, perfectly aligned reads, and non-primary alignments. Mismatches, insertions, deletions, and base clipping may all be introduced to allow the alignment of reads onto the reference. To handle substitutions between reference and query, mismatches are introduced. Multiple base pair divergences are treated as insertions to the reference or as deletions from the reference. Base clipping masks portions of reads (from either end) that do not align to the reference from end to end, meaning shorter but contiguous read fragments are aligned. The extent of these operations in alignment can particularly characterize differences in alignments to linear references and population graphs. With the expectation that the incidence of these operations decreases in graph alignments (in proportion to the number of aligned bases).

The number of aligned and unaligned reads are indicative of the proportion that aligns in a read set. For instance reads may not be aligned at all because of insufficient sequence context on the reference or due to low read quality, random noise, and/or contamination. The number of bases that are aligned provide more detail, given that not all reads are perfectly aligned. Perfectly aligned reads, describe full length alignments of reads for which no mismatches/insertions/deletions/clipping are introduced. The number of bases that are unaligned includes the bases from unaligned reads, mismatches, insertions, and clipped bases. Reads for which there are multiple valid alignments that score equally, result in non-primary alignments. Meaning that for every read there will always be a primary alignment (or it is unaligned), and one or more non-primary alignments. The incidence of these non-primary alignments give an indication on the extent of ambiguity in the alignments, given that this is typically induced by repetitivity in the reference.

5 Constructing a population graph from known variants

One way of constructing population graphs, is projecting sets of variations (from VCF files) called with respect to a reference genome back onto this reference (Similar to construction in others methods such as vg and Graphtyper) (Fig. S3a). Initially a singleton graph is created, which encodes the reference sequence (Fig. S3b). Variants are, according to their reference coordinate ordering, iteratively inserted in the graph. For each variant, a minimum of three nodes is introduced into the graph. The reference node is first split into two nodes, describing sequence before and after the variation. Between these reference nodes the reference and alternate alleles are introduced (Fig. S3c). In case of consecutive variants (variations at most one base-pair apart), the reference and variant alleles are connected to the preceding nodes and only then converge into a reference node (Fig. S3d). The same procedure applies for both indels and SNPs (Fig. S3e). Haplotyping information is embedded on the edges, which includes the sample(s) and the reference.

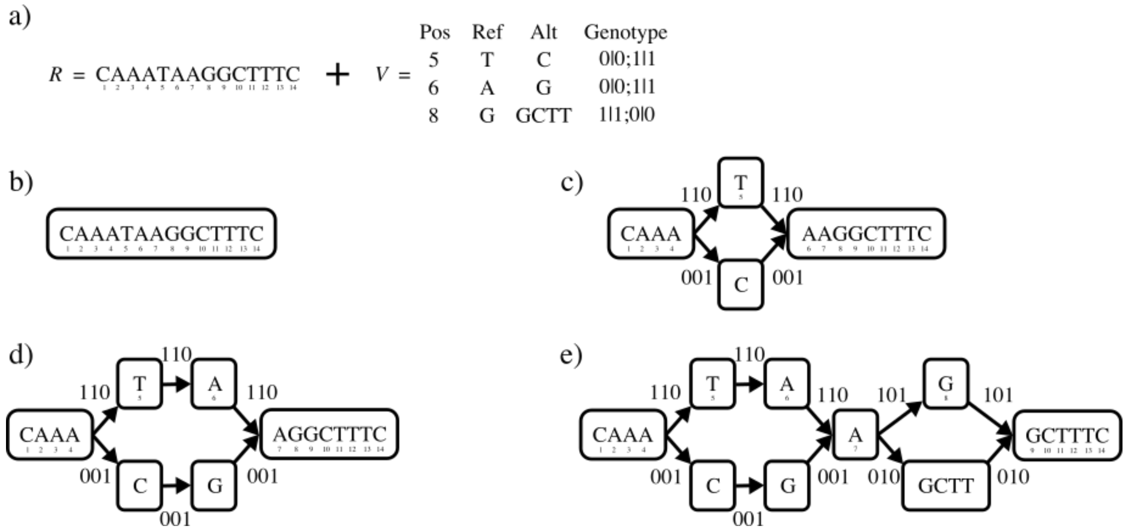

Fig. S3: a) The reference sequence and variation set used in graph construction. b) A graph is initialized with a single node encoding the reference sequence. c) In order of the reference coordinate space, the variant T → C is introduced into the graph. d) A consecutive variant (A → G) is added to the graph. e) An insertion (G → GCTT) is added to the graph. The final population graph encodes three paths, one of which is the reference path.

The described graph construction strategy of CHOP differs to that of vg. The most notable change being the full combination of (consecutive) alleles and treatment of SNPs and indels, as is shown in Fig. S4 for CHOP and vg.

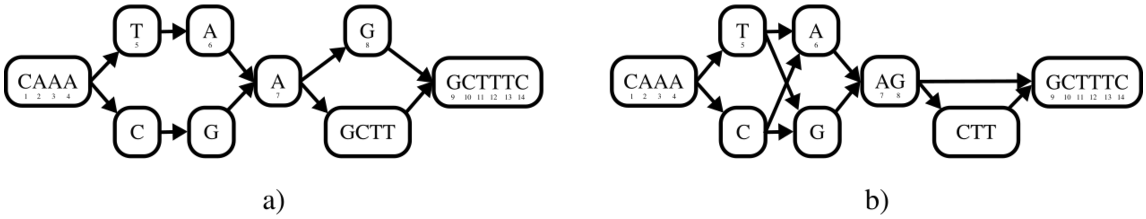

Fig. S4: Graph construction with the same input genome and variants as in Fig. S3. a) Graph construction using CHOP. b) Graph construction with vg construct.

Because the graph construction methods of CHOP and vg are similar but not entirely the same this may also affect indexing (see Fig. S5) resulting in a different number of indexed paths in CHOP and vg respectively.

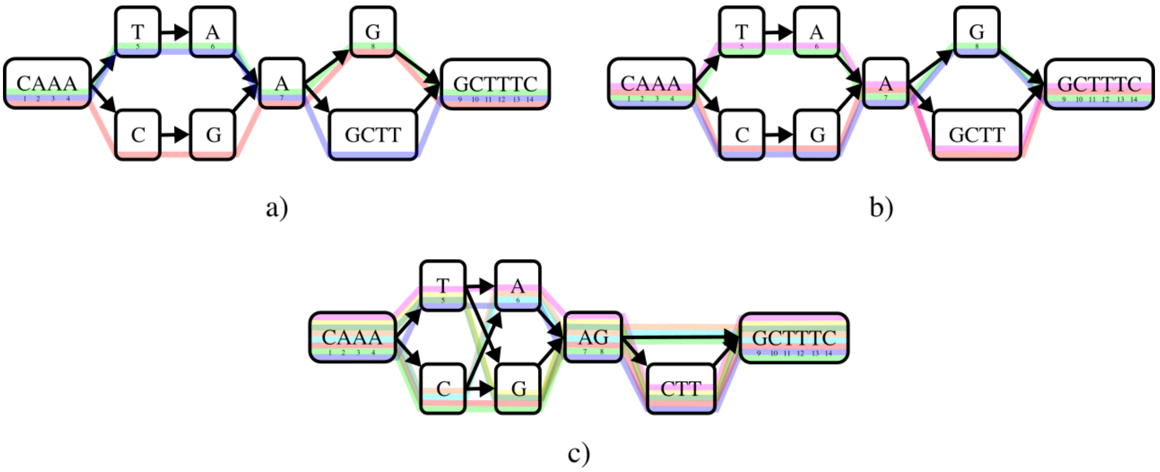

Fig. S5: The graph construction strategy of CHOP and vg (details in Fig. S4) can affect the resultant paths that are indexed. a) CHOP extracts three paths from the CHOP constructed graph. b) If the same graph is indexed by vg, there will be four paths. c) The vg constructed graph indexed by vg has eight paths.

We evaluated whether read alignment is affected by the two differently constructed graphs. To do so, we aligned reads from sample SRR833154 with vg to graphs constructed by both vg and CHOP during the MTB hold-out experiment. For each of these alignments we took the ratio from the counted number of perfectly aligned reads, unaligned reads, and mismatches. The ratio is calculated by dividing (for example) the number of mismatches counted in the vg constructed graph alignment by those counted in the CHOP constructed graph alignment. Therefore, if there would be no difference between the methods the ratio should be equivalent to 1.0. The results in Fig. S6 show that there is minimal difference.

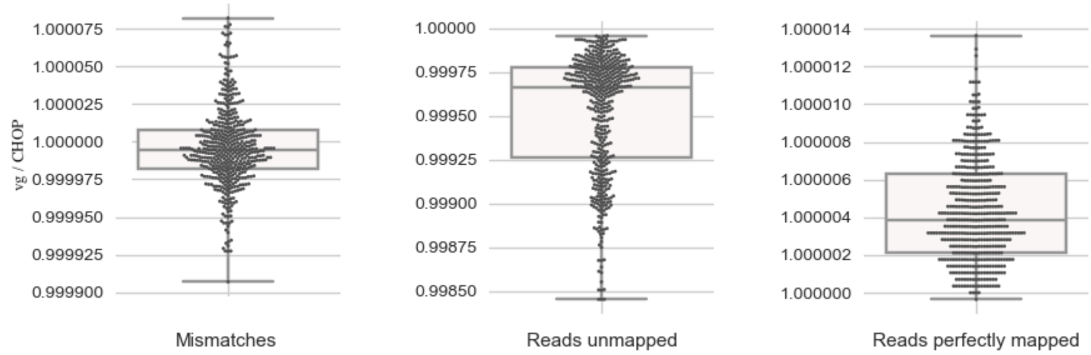

Fig. S6: SRR833154 graph alignments with vg using graphs constructed by either CHOP or vg. The y-axis represents a ratio (vg over CHOP) for the number of mismatches, unaligned reads, and perfectly aligned reads counted in the alignments.

6 *Mycobacterium tuberculosis* read alignment to H37Rv and graph

In the hold-out experiment 10 different single-end read sets are aligned to the reference genome, H37Rv, and to population graphs that progressively include more samples (up to 400 excluding the hold-out). Alignments were evaluated on both a read and base-count basis. Shown for SRR833154 this includes the number of perfectly aligned reads (Fig. 2), unaligned reads (Fig. S7), and mismatched bases (Fig. S8).

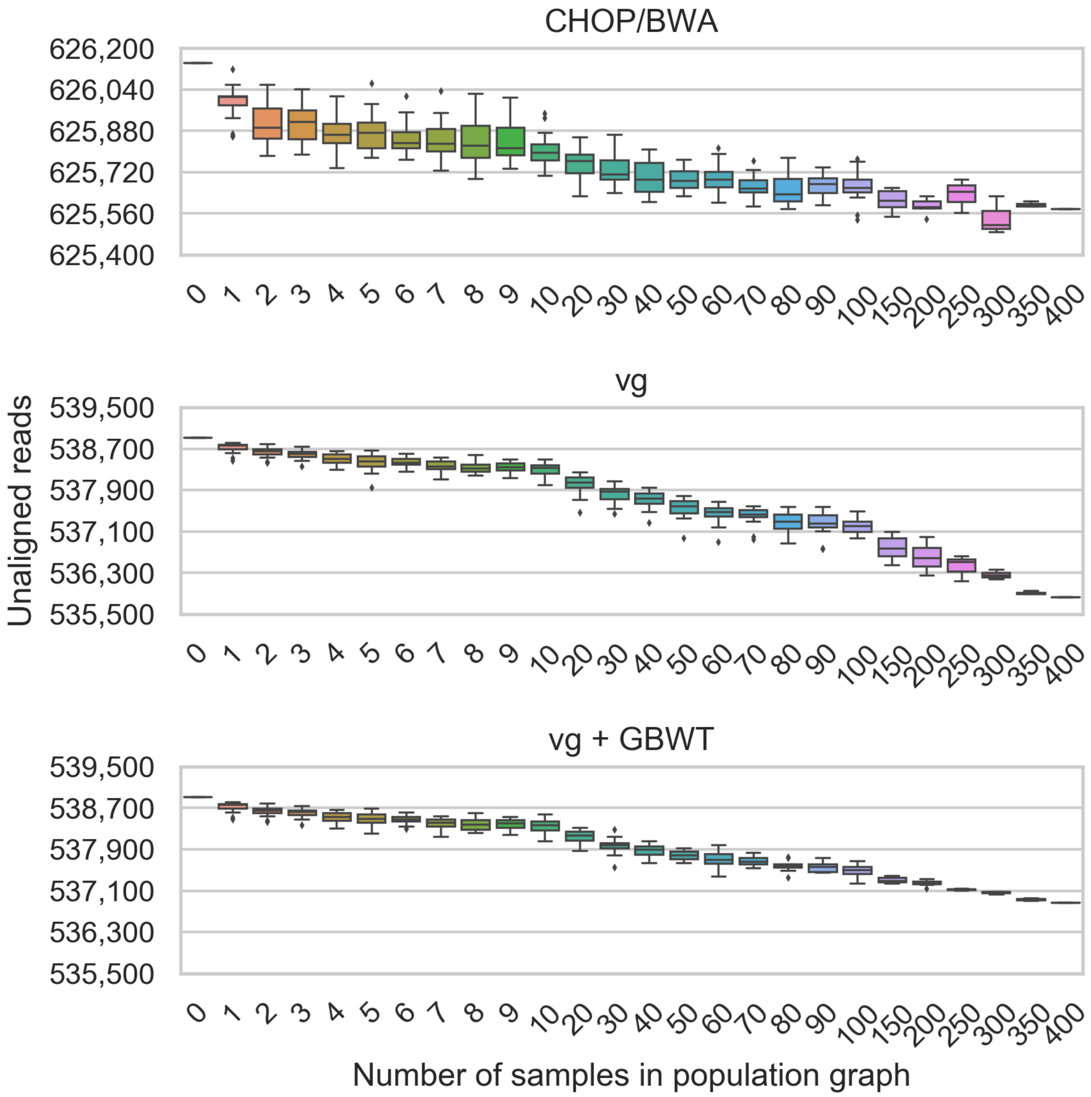

Fig. S7: Unaligned read count for SRR833154 alignments to different sized population graphs, containing between 0 (only H37Rv the linear reference) and 400 samples.

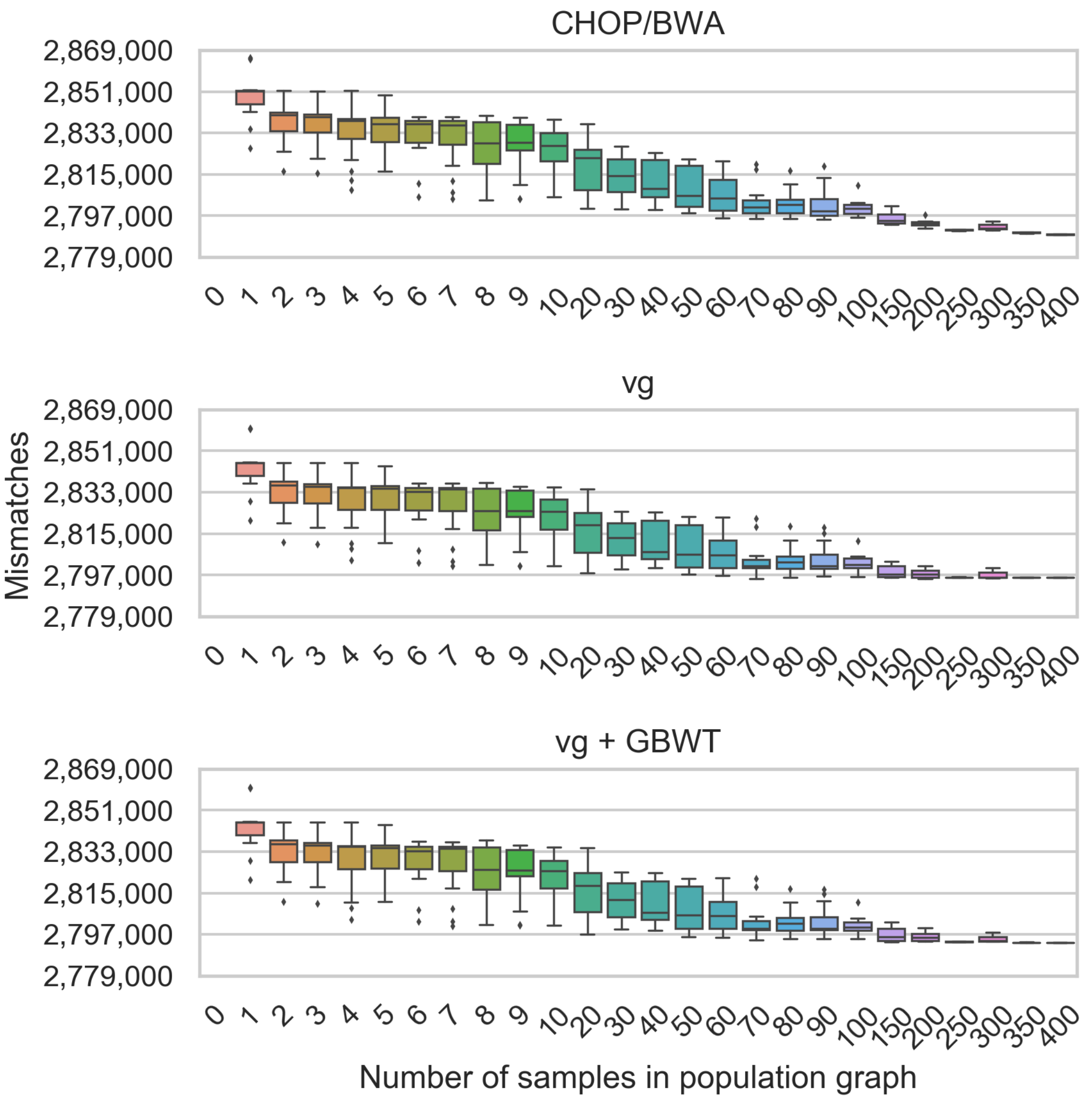

Fig. S8: Mismatch base count for SRR833154 alignments to different sized population graphs, containing between 0 (only H37Rv the linear reference) and 400 samples.

7 Aligning to CHOP null graphs with vg

One strategy to directly compare CHOP to vg+GBWT and exclude any aligner specific differences (BWA and vg), is to use the CHOP null graphs with vg. When considering the null graph, each node within can be understood as a path in the corresponding population graph, however these paths can also be considered to be disjoint subgraphs. Interoperability of CHOP and vg should therefore be possible if we consider the null graph to be a collection of disjoint subgraphs. Since vg has its own subroutines to build graphs, we were able to directly construct vg graphs from the null graphs we generated.

We initially evaluated this setup (denoted as CHOP/vg) using the same 10 MTB graphs (n=400, with one sample as a hold-out to align with). Here CHOP was run for $k = 101$ on each of these graphs, with 16,909 paths being encoded on average within the null graphs. When converting the null graphs into the vg format the graphs were ~9% larger on disk compared to when we build these graphs from a supplied FASTA and VCF file (with construction time being approximately the same). However, indexing the graphs with vg (GCSA2 and xg) took substantially longer (average 2,558 seconds), which is ~16x slower compared to indexing the VCF constructed graphs (average 164 seconds). For completeness, we also attempted to index the 1000 Genomes chromosome 6 null graph with vg. However, we were not able to index the graph, as indexing time exceeded more than 7 days (for reference this indexing took 5,751 and 33,619 seconds for vg and vg+GBWT respectively).

In Table S2, we summarize the same alignments results as in Table 1, which now includes the alignment results of CHOP/vg. Overall, the results between CHOP/vg and vg+GBWT are similar. However, there is a 5x increase in the number of non-primary alignments, which appears to be the result of increased redundancy in the (CHOP generated) index. This may explain the slower index construction time as well as increased alignment times. Taken together these results further confirm that the alignment results of CHOP and vg are similar, certainly when the same aligner is being used. Consequently, differences between the two haplotype-aware aligners are predominantly in how the haplotype constraints are implemented (fundamentally as in CHOP, or by the combined effort of GCSA2 and GBWT indexing in vg) as well as in the efficiency of the aligner (BWA or vg).

Table S2: Mean of alignment results across all 10 hold-out sample alignments to 1) the reference genome H37Rv (H37Rv columns) and 2) the 400 MTB genomes graph (Graph columns) for CHOP/BWA, vg with and without haplotyping, and CHOP/vg to align the reads (note that when aligning only to H37Rv, CHOP is not used).

	All TB hold-out samples - Read length = 101					
Alignment criteria	BWA	CHOP/BWA	vg	vg	vg + GBWT	CHOP/vg
	H37RV	Graph (n=400)	H37RV	Graph (n=400)	Graph (n=400)	Graph (n=400)
Reads aligned	6,160,920	6,162,033 (+0.018%)	6,241,270	6,245,907 (+0.074%)	6,244,004 (+0.044%)	6,243,852 (+0.041%)
Reads unaligned	360,236	359,123 (-0.309%)	279,886	275,249 (-1.657%)	277,152 (-0.977%)	277,304 (-0.922%)
Reads perfectly aligned	4,048,774	4,142,052 (+2.304%)	4,048,774	4,153,217 (+2.580%)	4,153,124 (+2.577%)	4,152,800 (+2.569%)
Bases aligned	596,380,132	596,611,260 (+0.039%)	599,244,753	599,601,399 (+0.060%)	599,528,267 (+0.047%)	599,536,504 (+0.049%)
Bases unaligned	62,191,423	61,960,355 (-0.372%)	59,307,655	58,949,429 (-0.604%)	59,023,102 (-0.480%)	59,014,752 (-0.494%)
Bases unaligned from clipped reads	22,349,569	22,380,472 (+0.138%)	27,442,533	27,690,552 (+0.904%)	27,575,464 (+0.484%)	27,548,619 (+0.387%)
Bases mismatched	3,458,029	3,308,480 (-4.325%)	3,596,667	3,458,707 (-3.836%)	3,455,296 (-3.931%)	3,458,399 (-3.844%)
Bases inserted	65,210	65,151 (-0.090%)	84,358	85,938 (+1.874%)	85,397 (+1.232%)	85,510 (+1.366%)
Bases deleted	52,272	51,165 (-2.118%)	70,324	72,082 (+2.500%)	70,347 (+0.033%)	70,659 (+0.476%)
Non-primary alignments	246,092	246,540 (+0.182%)	539,309	724,904 (+34.414%)	724,613 (+34.360%)	**3,585,924 (+564.911%)**
Time (seconds)	533	721	10,711	4,457	4,540	**5,534**

8 Comparing CHOP/BWA to HiSat2

HiSat2 [3] bases their index on the GCSA index [8], the precursor of the GCSA2 index used in vg, and then creates a global graph FM index along with multiple smaller region-specific graph FM indexes. We used the latest stable release of HiSat2 v2.1.0. Our attempts to index the 1000G chromosome 6 graph with HiSat2 failed, with memory usage exceeding 200 GB within 709 seconds. We believe this to be a result of exponential growth in the number of k-paths in the graph. Unfortunately, there is no parameter (as in vg) to tune path lengths, so we were not able to evaluate the alignment performance of this graph.

```
$ hisat2-build /.../hs37d5_chromosome_GRCh37_6_1_171115067_1.
   ↪ fa --large-index \
--snp /.../ALL.chr6.
   ↪ phase3_shapeit2_mvncall_integrated_v5_extra_anno
   ↪ .20130502.genotypes.snp \
hs37d5_chromosome_GRCh37_6_1_171115067_1
```

Therefore, we switched our attention to the MTB graphs (n=400 samples, plus the reference genome). Indexing these graphs took approximately 131 seconds and 12 GB of memory for each graph. The memory footprint of HiSat2 on these graphs is considerably higher than any of the tested methods.

In Table S3, we summarize the alignments results. Although indexing is costlier, HiSat2 aligns faster than BWA, CHOP/BWA, and vg. There are however great differences in the alignment statistics. The HiSat2 aligner has far more unaligned reads in both the baseline and the graph alignments. This can be understood as an aligner specific difference, i.e. the aligner is less sensitive than BWA or vg. However, what is surprising is that the number of unaligned reads increases when considering the alignment to the graph with respect to the linear genome. If there would be an increase in non-primary alignments this could be attributed to multi-mapping reads, however the number of non-primary alignments actually decreases (we did not observe this in CHOP or vg), suggesting a different cause. We speculate that there might be shortcomings in the full representation of all haplotypes leading to missing sequence, which would explain both the increased number of unaligned reads and decreasing non-primary alignments. From these experiences, we conclude that HiSat2 does not scale well and that the alignment results are not in agreement with those of CHOP/BWA and vg.

Table S3: Mean of alignment results across all 10 hold-out sample alignments to 1) the reference genome H37Rv (H37Rv columns) and 2) the 400 MTB genomes graph (Graph columns) for CHOP/BWA (note that when aligning only to H37Rv, CHOP is not used), vg with and without haplotyping, and HiSat2 to align the reads.

All TB hold-out samples - Read length = 101

Alignment criteria	BWA	CHOP/BWA	vg	vg	vg + GBWT	HiSat2	HiSat2
	H37RV	Graph (n=400)	H37RV	Graph (n=400)	Graph (n=400)	H37RV	Graph (n=400)
Reads aligned	6,160,920	6,162,033 (+0.018%)	6,241,270	6,245,907 (+0.074%)	6,244,004 (+0.044%)	5,536,194	**5,489,149 (-0.850%)**
Reads unaligned	360,236	359,123 (-0.309%)	279,886	275,249 (-1.657%)	277,152 (-0.977%)	984,962	**1,032,007 (+4.776%)**
Reads perfectly aligned	4,048,774	4,142,052 (+2.304%)	4,048,774	4,153,217 (+2.580%)	4,153,124 (+2.577%)	4,056,850	4,113,818 (+1.404%)
Bases aligned	596,380,132	596,611,260 (+0.039%)	599,244,753	599,601,399 (+0.060%)	599,528,267 (+0.047%)	553,338,901	**548,757,802 (-0.828%)**
Bases unaligned	62,191,423	61,960,355 (-0.372%)	59,307,655	58,949,429 (-0.604%)	59,023,102 (-0.480%)	105,271,191	**109,852,201 (+4.352%)**
Bases unaligned from clipped reads	22,349,569	22,380,472 (+0.138%)	27,442,533	27,690,552 (+0.904%)	27,575,464 (+0.484%)	3,625,336	3,589,573 (-0.986%)
Bases mismatched	3,458,029	3,308,480 (-4.325%)	3,596,667	3,458,707 (-3.836%)	3,455,296 (-3.931%)	2,164,703	2,029,911 (-6.227%)
Bases inserted	65,210	65,151 (-0.090%)	84,358	85,938 (+1.874%)	85,397 (+1.232%)	26,674	26,763 (+0.334%)
Bases deleted	52,272	51,165 (-2.118%)	70,324	72,082 (+2.500%)	70,347 (+0.033%)	11,793	11,756 (-0.317%)
Non-primary alignments	246,092	246,540 (+0.182%)	539,309	724,904 (+34.414%)	724,613 (+34.360%)	969,452	**755,436 (-22.076%)**
Time (seconds)	533	721	10,711	4,457	4,540	312	517

9 Variation linkage of 1000 Genomes in chromosome 6

The 1000 Genomes Phase 3 variant set of chromosome 6 encodes 5,023,970 variants. In order to determine how much of the variation was shared among samples, we evaluated the genotyping of every variant as is shown in Fig. S9. This revealed that 41.58% of all variants are unique to its sample.

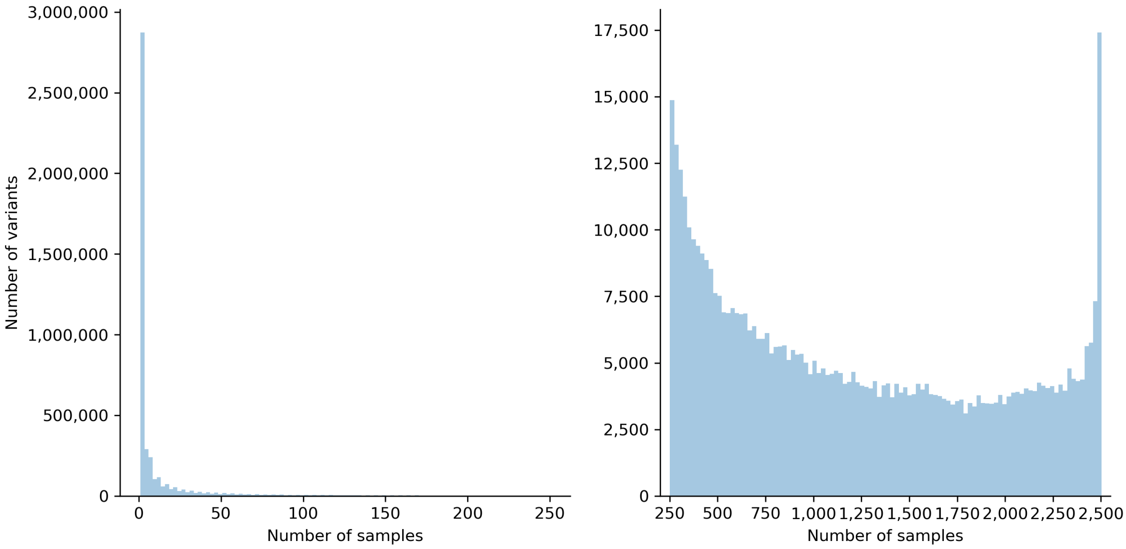

Fig. S9: Two histograms displaying the extent of shared variations among samples in the 1000 Genomes Phase 3 data.

10 Filtered 1000 Genomes read sets

Since we align only to a graph of chromosome 6, the single-end read sets from the 1000 Genomes Project phase 3 (Table S4) were first filtered to exclude any reads aligning to other chromosomes. This was accomplished by aligning all 15 read sets to the human genome (excluding mitochondrial DNA) using BWA, and subsequently generating new read sets by extracting reads that were aligned to either chromosome 6 or those that were unaligned. We chose random populations and for each population chose two random samples (except for YRI for which we randomly picked one).

Table S4: The read sets from the 1000 Genomes phase 3 used in alignments to chromosome 6.

			Filtered reads		
Population	Sample	Read set	Count	Mapped to Chr6	Unmapped
ESN	HG02938	ERR257960	6,238,375	5,572,661 (89.33%)	665,714 (10.67%)
ESN	HG03521	ERR257962	6,012,874	5,252,933 (87.36%)	759,941 (12.64%)
FIN	HG00308	ERR050084	3,882,577	2,814,118 (72.48%)	1,068,459 (27.52%)
FIN	HG00380	ERR050085	4,234,048	3,280,314 (77.47%)	953,734 (22.53%)
GBR	HG01791	ERR052834	3,066,482	2,358,096 (76.90%)	708,386 (23.10%)
GBR	HG01789	ERR052836	3,454,819	2,664,211 (77.12%)	790,608 (22.88%)
GIH	NA20881	ERR068420	2,278,409	2,015,089 (88.44%)	263,320 (11.56%)
GIH	NA20884	ERR068423	1,765,696	1,545,396 (87.52%)	220,300 (12.48%)
IBS	HG01670	ERR050090	1,115,839	859,298 (77.01%)	256,541 (22.99%)
IBS	HG02223	ERR056986	1,808,334	1,467,485 (81.15%)	340,849 (18.85%)
KHV	HG01595	ERR059932	1,217,726	1,059,586 (87.01%)	158,140 (12.99%)
KHV	HG02017	ERR059937	1,375,752	1,205,911 (87.65%)	169,841 (12.35%)
MSL	HG03054	ERR251326	4,720,003	4,293,293 (90.96%)	426,710 (9.04%)
MSL	HG03378	ERR251401	3,650,563	3,263,964 (89.41%)	386,599 (10.59%)
YRI	NA18517	ERR239432	569,541	478,520 (84.02%)	91,021 (15.98%)

11 Reads aligning to mitochondrial DNA

Fig. S10 displays the distribution of the number of aligned bases for reads that aligned exclusively onto the graph. Of the reads corresponding to the peak at 69 bp as shown in Fig. S10, 97.54% of them aligned to the same fragment of a path in the graph. We used BLAST [1] to determine the origin of the path and found hits on chromosome 6 and mitochondrial DNA (corresponding to the fragment of the path). Realigning the same reads to mitochondrial DNA revealed that most of the reads align full length (100M), as is partially shown in the pileup of Fig. S11.

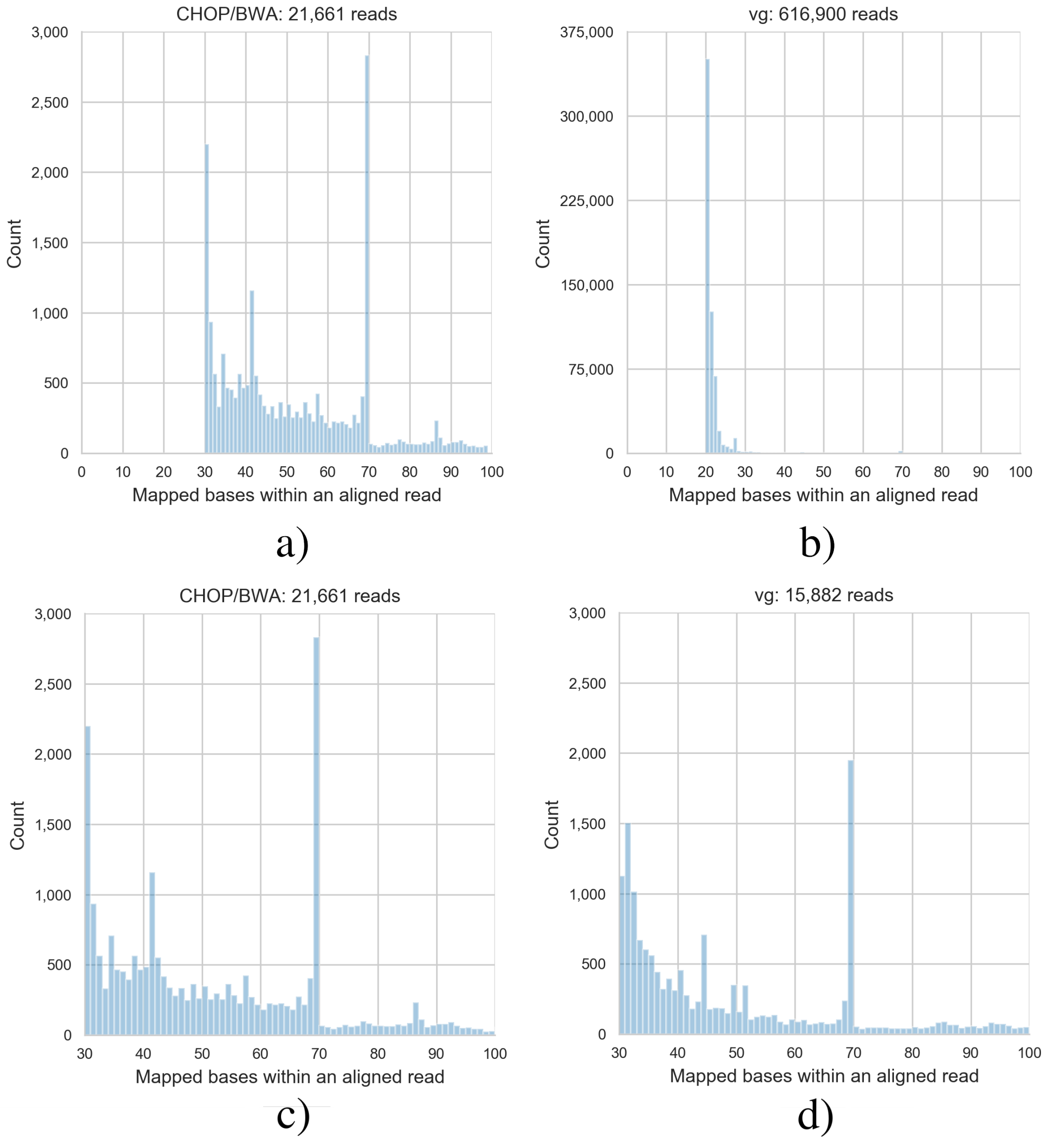

Fig. S10: The number of reads that have a particular number of bases aligned after their alignment onto the chromosome 6 population graph with CHOP/BWA (a) and vg (b), respectively. In c) and d) the same is shown in the range of 30 to 100 bases.

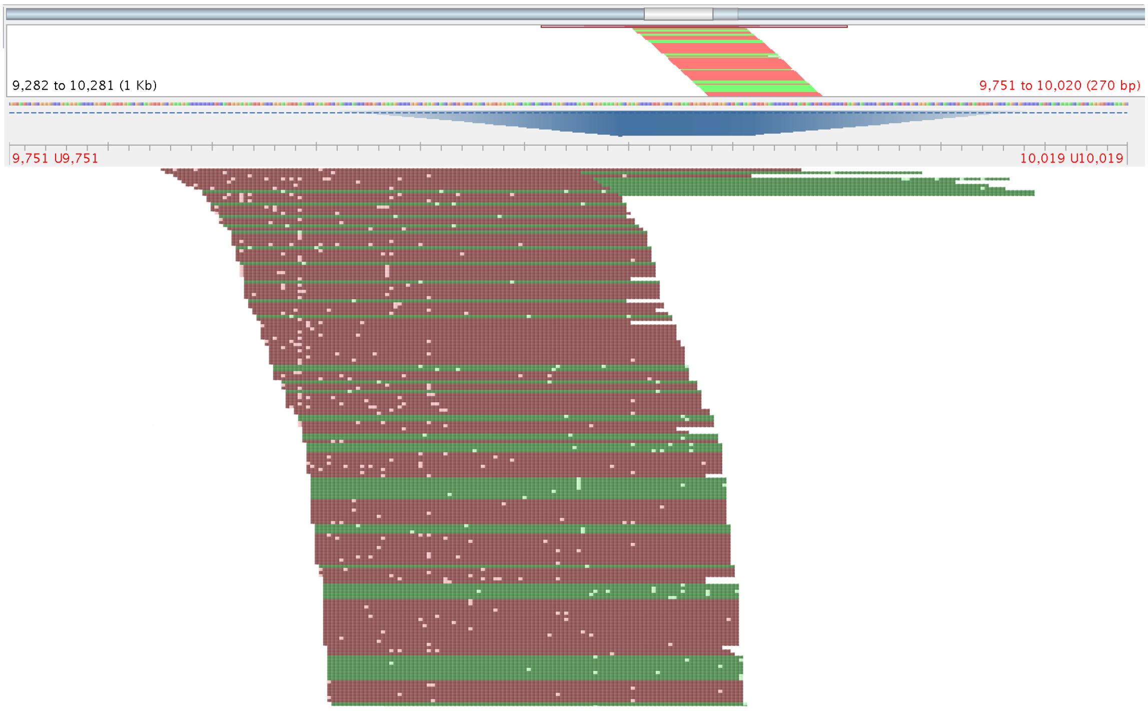

Fig. S11: Pileup visualization of mitochondrial DNA using Tablet [5] of reads that were previously unaligned on the linear reference genome (excluding mitochondrial DNA) but aligned on the graph that actually correspond to mitochondrial DNA.

12 Alignment accuracy in chromosome 6

To measure the accuracy of alignments generated by CHOP/BWA, we compared alignments of simulated reads to multiple linear references and graph-based references. Reads were simulated using Mason 0.1.2 [2], which includes sequencing errors and base calling quality, as well as annotations denoting the ground truth location of each simulated read. Alignments are noted as correct if the aligned read is within 1 bp of the ground truth position. Only primary alignments were considered. We simulated 10,000,000 sequencing reads from a chromosome 6 sequence that encoded variants of one sample with ID NA12878. Consequently, we generated a variation set encoding only SNPs, and excluded variations and genotyping specific to NA12878 and family members.

The simulated reads were aligned to the reference chromosome 6 to provide a baseline measurement of the accuracy, as well as to a personalized chromosome 6 (linear reference including all of NA12878's SNPs) to obtain an idealized situation. Three graphs were constructed from the NA12878 filtered variation set: Full; graph encoding all 1000G variation in chromosome 6 (excluding NA12878), Min2; graph encoding only variations that were observed in at least two individuals; PopCov10+; graph encoding the top 10% scoring variations as scored by FORGe [6], which weighs variants by allele frequency in the population and minimizes graph complexity. Fig. S12a shows the fractions of reads that are correctly and incorrectly aligned onto the different reference genomes. In Fig. S12b the sensitivity metrics of perfectly aligned reads and number of mismatches are shown for the same alignments.

Although alignment sensitivity rises as more variants are introduced into the population graphs, it also increases sequence repetitivity in the graph, which negatively influences alignment accuracy. This can be observed for both the Min2 and Full graphs which are less accurate than the baseline, while they have comparable sensitivity with respect to the idealized reference genome. The trade-off of sensitivity and specificity is clearly seen when variant selection is performed, as is the case with the PopCov10+ graph, which improves accuracy, at the cost of sensitivity.

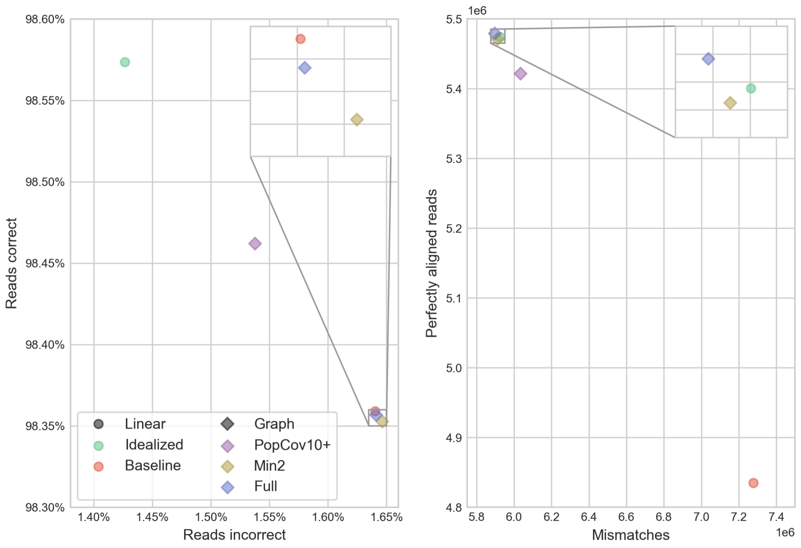

a) b)

Fig. S12: Alignment statistics of the NA12878 simulation. a) The fraction of correctly aligned reads and incorrectly aligned reads. b) Sensitivity metrics of perfectly aligned reads and number of mismatches.

13 Aligning to a graph encoding the MHC

Thus far we aligned to a variation graph (14,744,119 nodes and 19,770,411 edges) of chromosome 6 with all variants from the 1000G phase 3 (5,023,970 variants). We made no exception to any variants included in this graph, regardless of their quality. Although, we observed a clear improvement in alignment measures, we know that since the MHC regions are more difficult to align to, we can expect that the variants that were called within this region using the linear reference to be of poorer quality than found elsewhere.

To evaluate improvements in read alignment within the MHC region, we build a graph from the GRC38 reference and MHC alternate alleles through multiple sequence alignment (MSA) using REVEAL [4]. This MHC graph is naturally much smaller than the previously build 1000G graph, with only 28,753 nodes and 40,032 edges. Since this graph was build using MSA it also incorporates larger structural variations, which is something that was not yet explored using the variation graph.

Similarly, as described in section 2.2, we ran CHOP on this graph for k=100, and aligned the 15 read-sets onto the linear sequence of chromosome 6 (GRC38) and the null graph of the MHC graph using BWA. As the graph only includes alternate sequence originating from the MHC region, any improvements of alignment can be attributed towards their inclusion. Our findings are summarized in Table S5 together with our previous results when aligning to the linear sequence of chromosome 6 GRC37 and the 1000G graph. The transition from GRC37 to GRC38 has clear advantages when it comes to improving alignability. We do see an improvement of aligned reads to this MHC graph with respect to the linear reference genome (more aligned reads, less unaligned reads), showing the benefit of aligning reads to a population graph containing well-established haplotypes. The improvement on the MHC graph is less than for the 1000G variation graph, although we have to remark that performance scores on the 1000G variation graph are averaged over the entirety of chromosome 6 and will be less beneficial for the MHC region.

Table S5: Mean of alignment results from 15 samples from the 1000 Genomes data when aligning to 1) the reference genome sequence of chromosome 6 (column GRC37), 2) the 1000G graph created from the 5,008 haplotypes, 3) the reference genome sequence of chromosome 6 (column GRC38), 4) the MHC graph generated from a MSA with the reference sequence and the MHC alternate alleles.

	1000 Genomes samples - Read length = 100			
Alignment criteria	BWA	CHOP/BWA	BWA	CHOP/BWA
	GRC37	Graph (n=2504)	GRC38	MHC Graph
Reads aligned	2,542,399	2,543,522 (+0.044%)	2,551,410	2,551,774 (+0.014%)
Reads unaligned	483,670	482,548 (-0.232%)	474,659	474,296 (-0.077%)
Reads perfectly aligned	1,794,564	1,977,952 (+10.219%)	1,826,614	1,835,375 (+0.480%)
Bases aligned	251,122,992	251,516,725 (+0.157%)	252,080,501	252,142,201 (+0.024%)
Bases unaligned	51,439,949	51,070,534 (-0.718%)	50,483,271	50,423,549 (-0.118%)
Bases unaligned from clipped reads	1,801,947	1,846,687 (+2.483%)	1,812,049	1,814,726 (+0.148%)
Bases mismatched	1,270,981	969,087 (-23.753%)	1,205,282	1,179,256 (-2.159%)
Bases inserted	43,979	19,661 (-55.296%)	43,148	41,170 (-4.584%)
Bases deleted	61,659	32,355 (-47.526%)	60,870	58,445 (-3.983%)

14 Variation detection with CHOP/BWA and Graphtyper

The main purpose of Graphtyper is to genotype variants using a population graph and realigning reads in an existing alignment. Unfortunately Graphtyper does not output a graph realignment of the input linear alignment, meaning we cannot evaluate its seeding and aligning capabilities. However, we can still compare its variant calling ability to CHOP/BWA. Since Graphtyper relies on a linear alignment (a BAM file from an aligner such as BWA), an implicit reference allele bias is introduced in the process. This bias should be reduced to some extent through the realignment of reads onto a given graph.

Note that Graphtyper has hardcoded limitations with regard to which genomes can be used with it. Because of this limitation only human genomes (GRC37 and GRC38) can be used with the tool. As such, we evaluated GraphTyper on human data with the variants from the 1000 Genomes Project Phase 3, using the latest stable release of Graphtyper v1.4. Our evaluations started with the 1000G chromosome 6 graph and the linear reference sequence of chromosome 6. Graph construction took 4,753 seconds (in the case of the graph) and 8 seconds (for the linear reference). Graph indexing took 16 GB memory, 8,248 seconds (graph), and 10 GB memory, 2,285 seconds (linear reference).

With this setup we aligned, with BWA, reads from ERR050084 (sample HG00308) (prefiltered to only include reads that align to chromosome 6 or that were unaligned elsewhere on the genome (3,882,577 reads)) onto the linear sequence of chromosome 6. While variant calling a population graph already lowers the number of newly called variants, this is even lower in this particular graph since the variants (as called on a linear reference) of HG00308 are encoded in the graph. We called variants and genotypes using "Graphtyper call". This took 126,456 seconds (1.4 day) and 33 GB peak memory with the linear graph. Next, we called variants on the 1000G graph (same command) which finished after 382,329 seconds (4.4 days), with peak memory at 93GB.

```
$ graphtyper call /.../graphtyper.gt --sam=/.../.bam --index
   ↪ =/.../.gt_gti 6
```

We followed the same steps for CHOP/BWA (note that in the linear case this reduces to running BWA only), variant calling was done using bcftools. Variant calling the linear genome took 700 seconds and 200 MB peak memory. The 1000G graph finished after 1,620 seconds (0.45 hours), with peak memory at 9.5 GB.

```
$ bcftools mpileup --redo-BAQ --min-BQ 30 --per-sample-mF --
   ↪ annotate DP,AD -f /.../ref.fa -O b /.../.bam | bcftools
   ↪  call --multiallelic- --variants-only -Ob > /.../out.
   ↪ bcf
```

We focus on variant calling, as this would allow us to compare CHOP/BWA to GraphTyper, especially since GraphTyper can also call new variants at realigned positions. Variant calling the linear graph with GraphTyper yielded no new variants (which was unexpected) or genotyped calls (as expected). It is not clearly defined how GraphTyper deals with linear graphs, as such we are not sure what to exactly expect in this scenario when it comes to calling new variants. The 1000G graph alignment yielded 0 new variant calls and 4,683,374 genotyped variant sites. The number of genotyped sites is extremely high relative to the number of variants (5M) encoded in the graph. It is clear that GraphTyper is highly sensitive and reports many false positive variants, hence filtering would be necessary to reduce this. The recommended procedure (as seen in the GraphTyper repository), was to utilize the vcffilter

tool (part of vcflib https://github.com/vcflib/vcflib). This reduced the number of genotyped variant sites to 144,800.

```
$ vcffilter -f "ABHet < 0.0 | ABHet > 0.30" -f "MQ > 30" -f
   ↪ "QD > 6.0" /.../.vcf
```

Variant calling the CHOP/BWA alignment yielded 142,979 variant sites for the linear genome, and 1,212 in the graph. As the utilized variant calling is highly sensitive, quality filtering reduced this number to 57 variants, originating from reads that were previously unaligned on the linear genome. These results are more in line with expectation: 1) the number of variants detected on the graph is considerably lower than on a single linear reference genome, and 2) the variants detected on the graph are on top of the specific haplotypes, containing already variants with respect to the reference genome, so that reads from these regions have a smaller chance to align to the reference genome (due to large variation). Taken together, we conclude from this experiment that CHOP/BWA is more time and memory efficient (being the main claims that we make), moreover, GraphTyper seems to generate unexpected results when variant calling.

15 Comparing CHOP/BWA to GraphAligner

GraphAligner [7] is a long read aligner for graphs and supports similar input and output procedures as vg, meaning we could directly input our vg constructed graph into GraphAligner. We aligned reads with GraphAligner (commit: 8e37ecbc832cca5538e8d1427803e313089b17fb) from the 15 samples on the 1000G chromosome 6 graph and linear chromosome 6 sequence.

```
$ GraphAligner -g /.../.vg -f /.../.fq.gz -a /.../.json -t 1
  ↪  -b 35 --try-all-seeds --seeds-mxm-cache-prefix ...
```

In Table S6 we summarize the results of the alignments. The measurements of GraphAligner come closer to that of CHOP/BWA and vg than HiSat2. Furthermore, alignment times are similar as those of CHOP/BWA. However, we note the same behavior as with HiSat2 wherein we see a drop in aligned reads (and increase in unaligned reads) when we align to the graph instead of the linear reference genome, which may be attributed to the optimizations for seeding in long reads rather than the short reads used here.

Table S6: Mean of alignment results from 15 samples from the 1000 Genomes data when aligning to 1) the reference genome sequence of chromosome 6 (column GRC37), and 2) the population graph created from the 5,008 haplotypes, for CHOP/BWA, vg with and without haplotyping, and GraphAligner.

	1000 Genomes samples - Read length = 100						
Alignment criteria	BWA	CHOP/BWA	vg	vg	vg + GBWT	GraphAligner	GraphAligner
	GRC37	Graph (n=2504)	GRC37	Graph (n=2504)	Graph (n=2504)	GRC37	Graph (n=2504)
Reads aligned	2,542,399	2,543,522 (+0.044%)	2,684,925	2,726,051 (+1.532%)	2,717,972 (+1.231%)	2,664,609	**2,630,670 (-1.274%)**
Reads unaligned	483,670	482,548 (-0.232%)	341,144	300,018 (-12.056%)	308,098 (-9.687%)	361,460	**395,399 (+9.389%)**
Reads perfectly aligned	1,794,564	1,977,952 (+10.219%)	1,807,158	1,993,967 (+10.337%)	1,993,469 (+10.310%)	1,789,327	1,950,435 (+9.004%)
Bases aligned	251,122,992	251,516,725 (+0.157%)	254,518,323	255,911,471 (+0.547%)	255,578,370 (+0.416%)	258,684,466	256,773,126 (-0.739%)
Bases unaligned	51,439,949	51,070,534 (-0.718%)	48,029,518	46,654,663 (-2.863%)	46,995,159 (-2.154%)	41,030,266	**43,649,514 (+6.383%)**
Bases unaligned from clipped reads	1,801,947	1,846,687 (+2.483%)	12,716,162	15,699,089 (+23.458%)	15,245,035 (+19.887%)	203,221	177,659 (-12.578%)
Bases mismatched	1,270,981	969,087 (-23.753%)	1,198,917	953,800 (-20.445%)	940,371 (-21.565%)	4,681,045	3,931,955 (-16.003%)
Bases inserted	43,979	19,661 (-55.296%)	59,078	40,786 (-30.962%)	33,391 (-43.480%)	2,541,719	1,925,093 (-24.260%)
Bases deleted	61,659	32,355 (-47.526%)	73,131	44,555 (-39.075%)	41,040 (-43.882%)	464,085	415,508 (-10.467%)
Time alignment (seconds)	544	1,807	19,996	10,436	10,871	916	2,102
Memory alignment (MB)	412	5,534	837	3,296	4,389	3,047	14,446
Time indexing (seconds)	186	CHOP: 43,625<br>BWA: 3,256	37	5,751	33,619	NA	NA
Memory indexing (MB)	245	CHOP: 56,969<br>BWA: 3,813	269	45,670	45,868	NA	NA

16 Effects of varying *k* size in CHOP

First, we want to address that exponential path evaluations are eliminated in CHOP because it utilizes haplotype information. At any position in a graph there can only be as many parallel paths as there are encoded haplotypes (this worst case scenario can only occur if none of the genomes in the graph share any linkage at this position). This was explored by changing the value of k in CHOP, i.e. the minimal k-mer for which an exact match is required. As k grows larger more variation is spanned, meaning more paths need to be explored in the graph (which should maximally be the number of haplotypes encoded in the graph).

We ran CHOP on a MTB graph ($n = 400$ samples, plus the reference genome), with values of k ranging from 1-10.000, and evaluated different metrics (Fig. S13). We observe an approximately linear growth in processing time, peak memory, and the total number of bases in the resultant null graph as the value of k increases. The number of nodes in the null graph decreases as k increases and indeed converges to the number of encoded genomes within the graph. From this we see that for this variation graph choosing a k larger than the largest recorded genome ($k > 4.4$ MB) results in a null graph with 401 nodes (400 VCF genomes and a single reference genome). For completeness generating such a null graph takes 559 seconds and encodes ~1.76 gigabases, which is equivalent to the concatenation of all genomes in the graph, with peak memory of 6.5 GB.

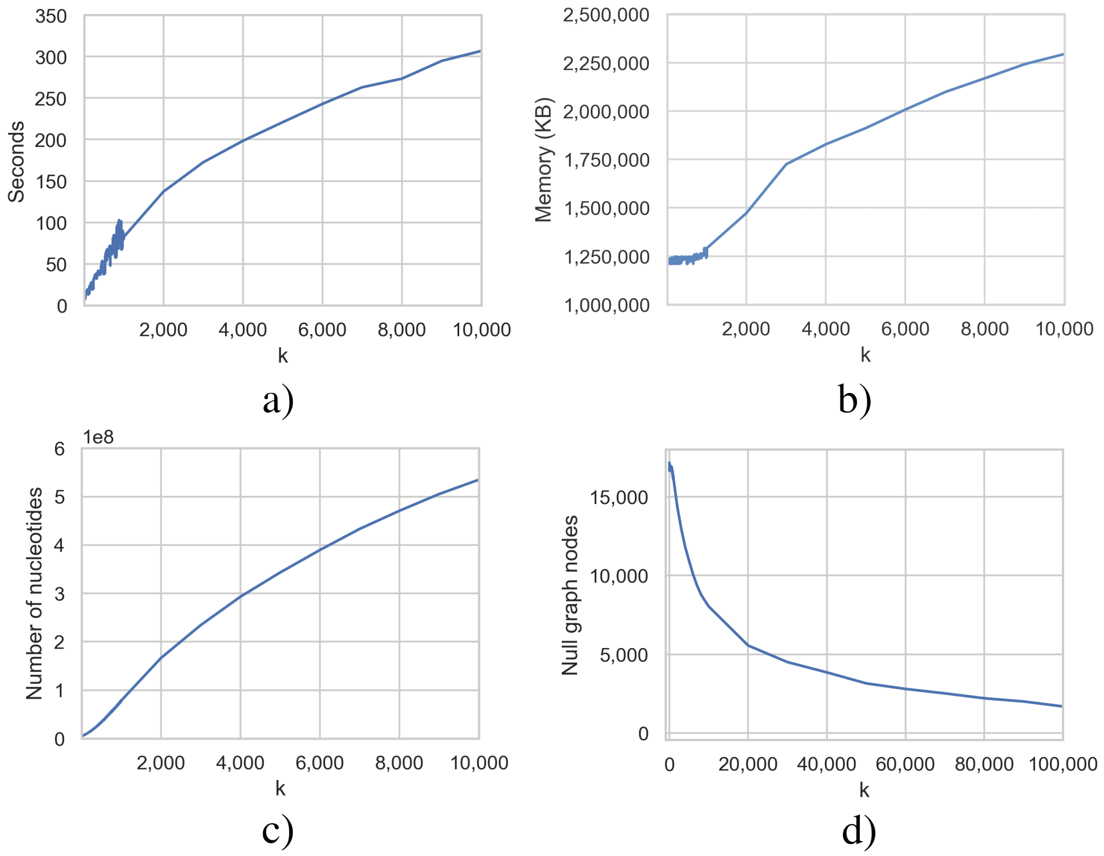

Fig. S13: Measurements of CHOP being run on a MTB graph (n=401) for increasingly larger values of k: a) indexing time; b) peak memory in KB; c) nucleotides encoded in the null graph; d) number of nodes in the null graph.

In Section 18 we show that there is a far higher density of variation in the 1000G chro-

mosome 6 graph. Due to this greater density, larger values for k will inevitably lead to an exponential explosion of paths. We observed such growth when indexing with vg(+GBWT) because of GCSA2 (note that we had to reduce k from 104 to 52 to be able to run vg(+GBWT)). This is prevented by CHOP. However, larger values for k will likely still lead to an intractable number of haplotype-constrained k-paths. To confirm this, we explored different settings of k for this graph, results are shown in Fig. S14. Indexing time grows — as expected — linearly. Peak memory, however, shows first a decrease and then an increase after which it changes linearly again. This erratic behavior might be caused by differences in the local densities of variation within the graph.

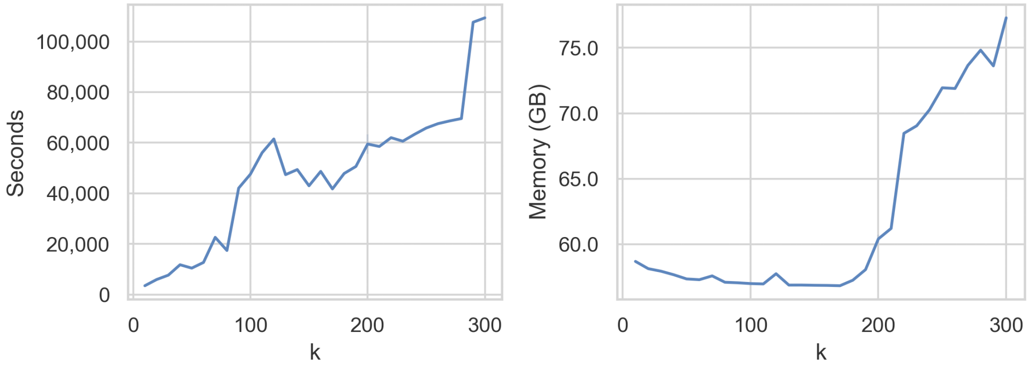

a) b)

Fig. S14: Measurements of CHOP being run on a population graph of human chromosome 6, for increasingly larger values of k: a) indexing time; b) peak memory in GB.

17 Simulated graph indexing

To better understand the behavior of CHOP under conditions of varying variation density, number of samples, and between sample variation linkage, we set up the following experiment using the H37Rv reference genome (4.4 MB) as a starting point. Given this reference we simulated sample VCFs with SNP variants uniformly distributed across the genome. In our simulation we used different variation groupings, simulating samples with exactly: 100, 500, 1000, 5.000, or 10.000 variants. For each of these groupings we also include variation linkage within the subsequent simulated sample given all previous observed samples. For example starting from a single simulated sample a, the subsequent simulated sample b will share some variation of the previous sample a, and the next simulated sample c will share this with both a and b. The amount of variation linkage represents another grouping, these include 5%, 10%, 20%, 30%, 40%, and 50% shared variation between samples. For each combination of grouping we then generated merged VCF files with a varying number of samples, this included 1 to 10 (step = 1), 10 to 100 (step = 10) and 100 to 500 (step = 100), i.e. 23 VCF files with different numbers of samples. Since these VCFs were generated for each combination of grouping we created 690 VCF files in total, Fig. S15 shows the variation distribution for each of these settings. We build graphs from each VCF file using the described VCF graph construction method. Subsequently, we ran both CHOP and vg+GBWT with $k = 104$ on each of the graphs and measured runtime and peak memory as shown in Fig. S16 and Fig. S17. Note that we allowed a maximum runtime of 4 hours and peak memory of 80 GB for each of the indexing methods.

The complexity of the graphs is highly variable and the majority can be indexed by both indexing methods within the set constraints (640 CHOP, 547 vg+GWBT). The remaining graphs were highly complex and encoded at least 5,000 (500 for vg+GBWT) variants per sample, which considering the reference genome results in regions very densely populated with variants. CHOP indexing is significantly faster, memory efficient, and able to handle more complex graphs than vg+GBWT. It is clear that increasing the between sample variation linkage simplifies the graph, with fewer unique variants being encoded, simplifying indexing for all approaches. Typically, variation can be expected to be shared at higher levels than shown here, especially when filtering out variants at low allele frequencies.

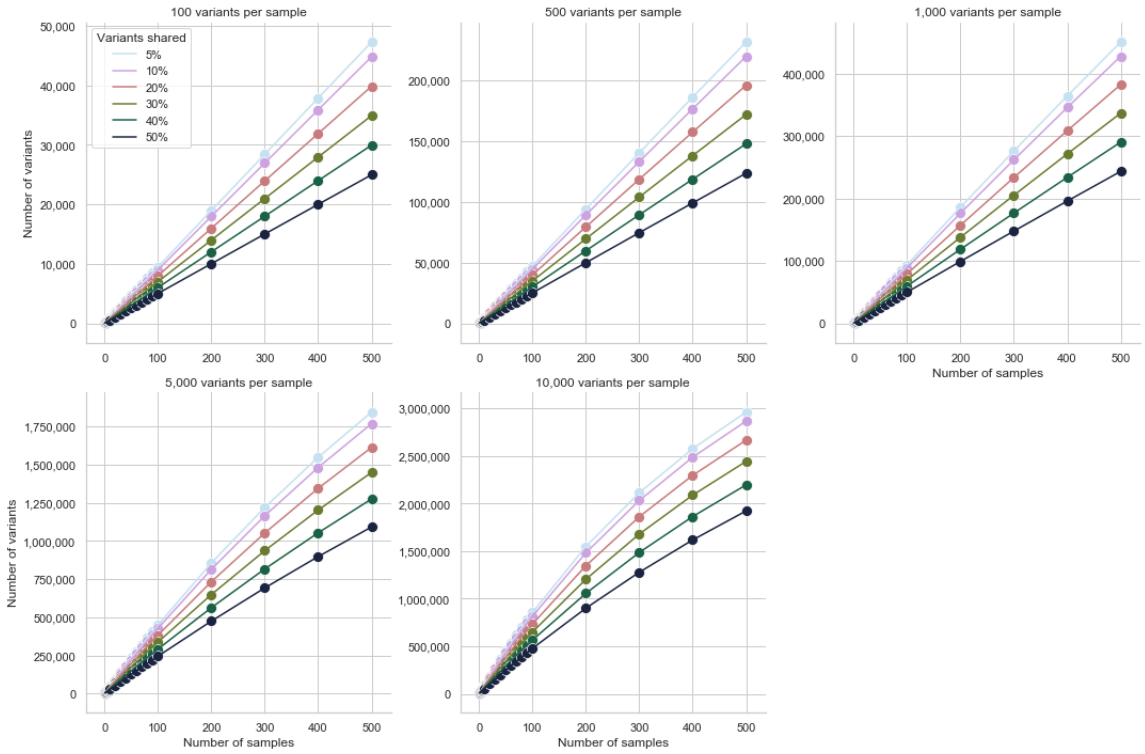

Fig. S15: The number of variants encoded in merged VCF files for all combinations of groupings: number of variants per sample (different plots) and probability of sharing variants with population (different colors).

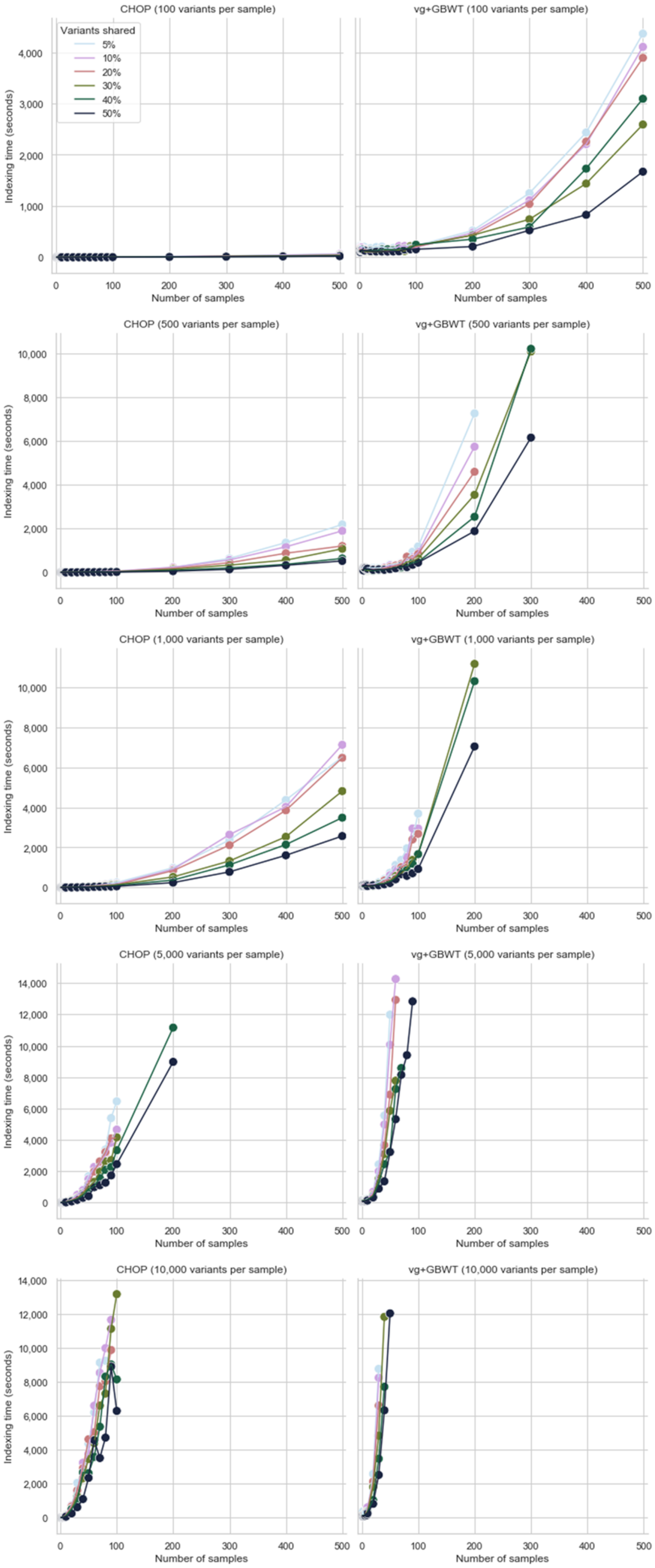

Fig. S16: CHOP and vg+GBWT indexing time (seconds) of the graphs, for all combinations of groupings: number of variants per sample and probability of sharing variants with population (different colors). Missing points in the plots indicate that indexing failed by exceeding one of the set constraints.

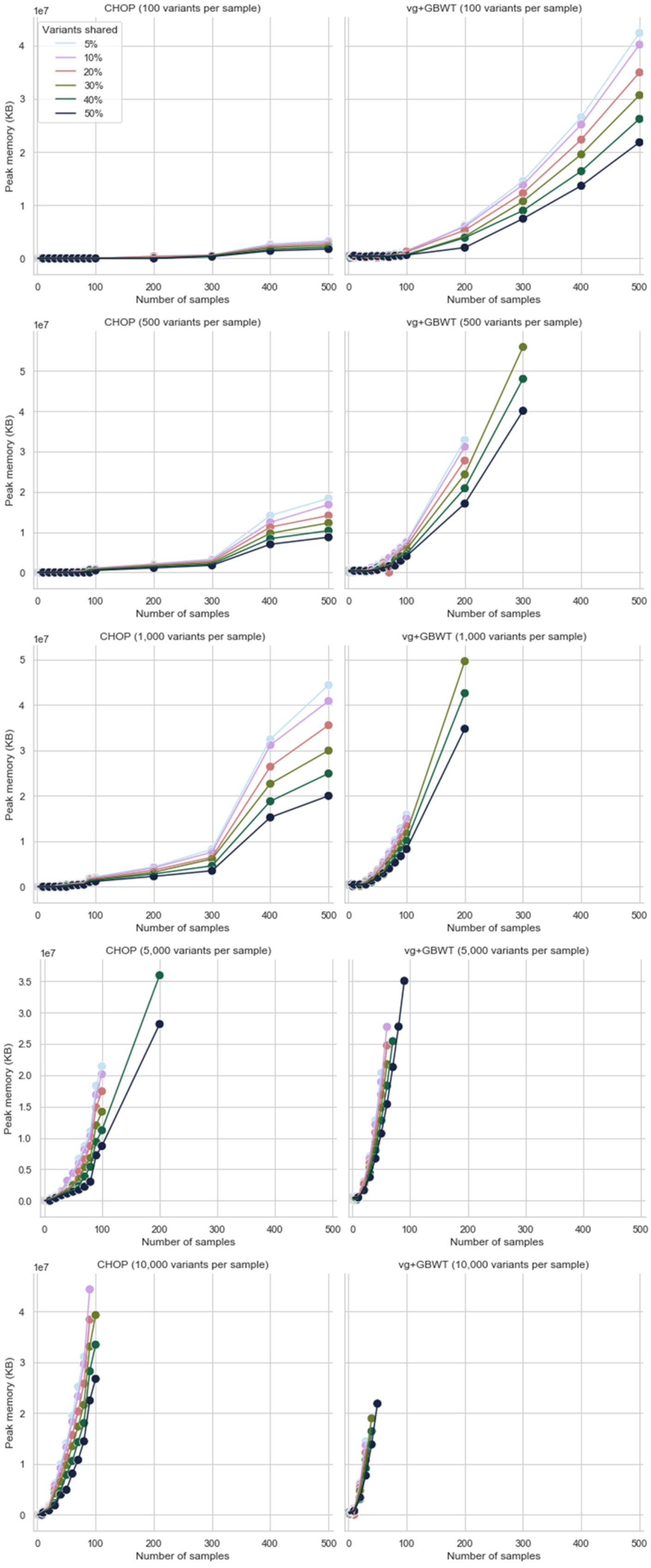

Fig. S17: CHOP and vg+GBWT peak memory (KB) during indexing of the graphs, for all combinations of groupings: number of variants per sample and probability of sharing variants with population (different colors). Missing points in the plots indicate that indexing failed by exceeding one of the set constraints.

18 Variant density in human chromosomes

The computational costs required to index human chromosomes is highly dependent on the number of variants encoded in the graph as well as the density of these variants and the chromosome size. For certain human chromosomes this variation density (possibly in combination with chromosome size) can lead to an explosive growth in the required memory/disk space, which is what happened with vg+GBWT for chromosomes 1, 2, 11, and X. To illustrate this, we quantified the number of variants across each chromosome in windows of 50 bp (note that we set vg to index $k = 52$ length paths) as is shown in Fig. S18. The chromosomes 1, 2, 11, and X each encode variants at higher densities than others, and chromosome 1 even exceeds 50 variants in a 50 bp window, note that these measurements should also be put into context of the chromosome size.

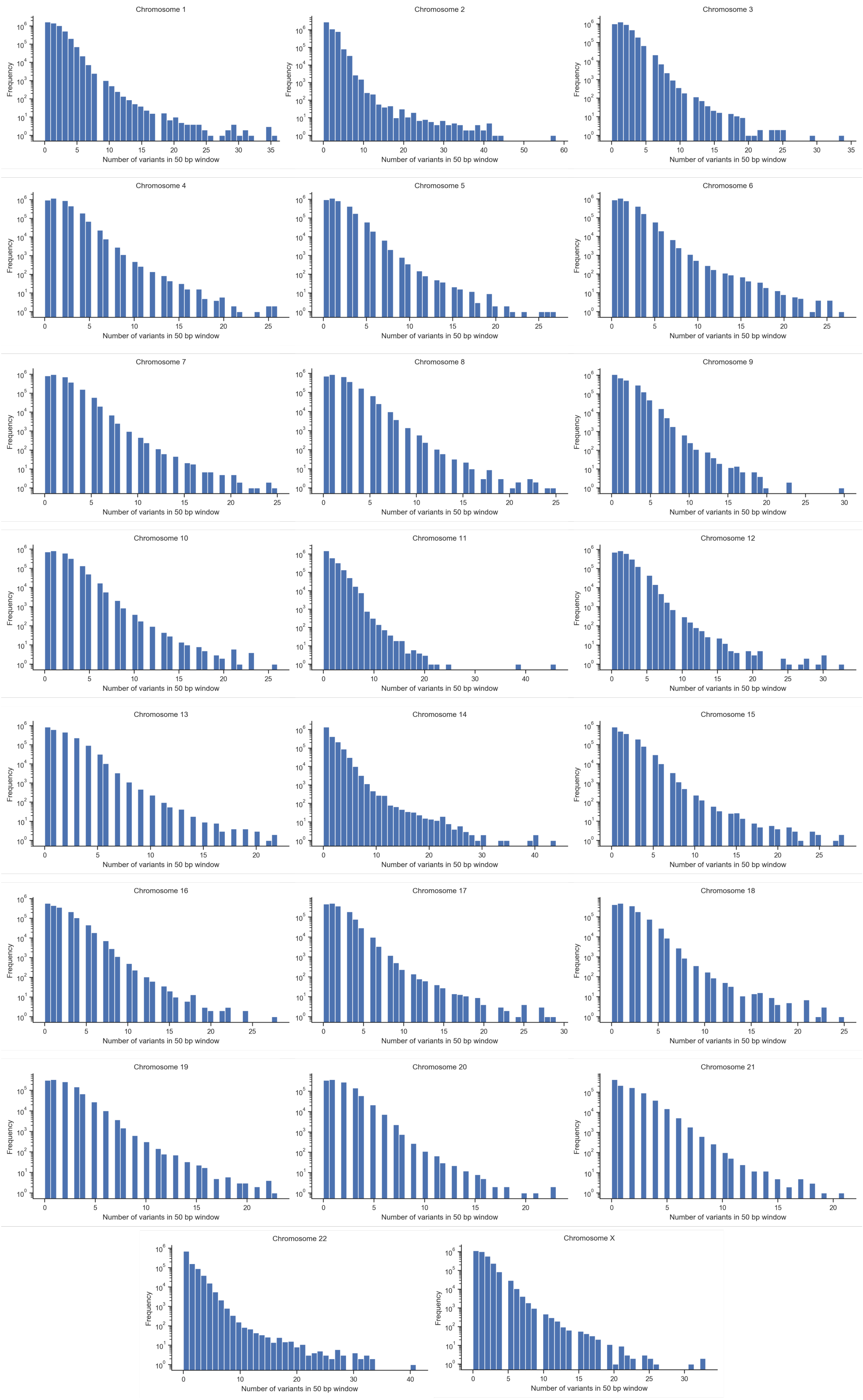

Fig. S18: Variant distribution in 50 bp windows for each human chromosome.

19 Variant integration

The realignment of SRR833154 reads onto a graph representation of H37Rv with variants detected from the alignment of SRR833154 reads onto H37Rv using CHOP/BWA, allows for the calling of novel variations using existing linear genome variant callers. Using Pilon we called variants in the graph alignment, of which 19 variants remained after quality filtering. Since, BWA by default outputs a SAM file, we can easily preprocess and prepare a BAM file that can be inspected using an alignment visualization tool such as Tablet [5]. In Fig. S19 we show newly aligned reads from which variants can be called.

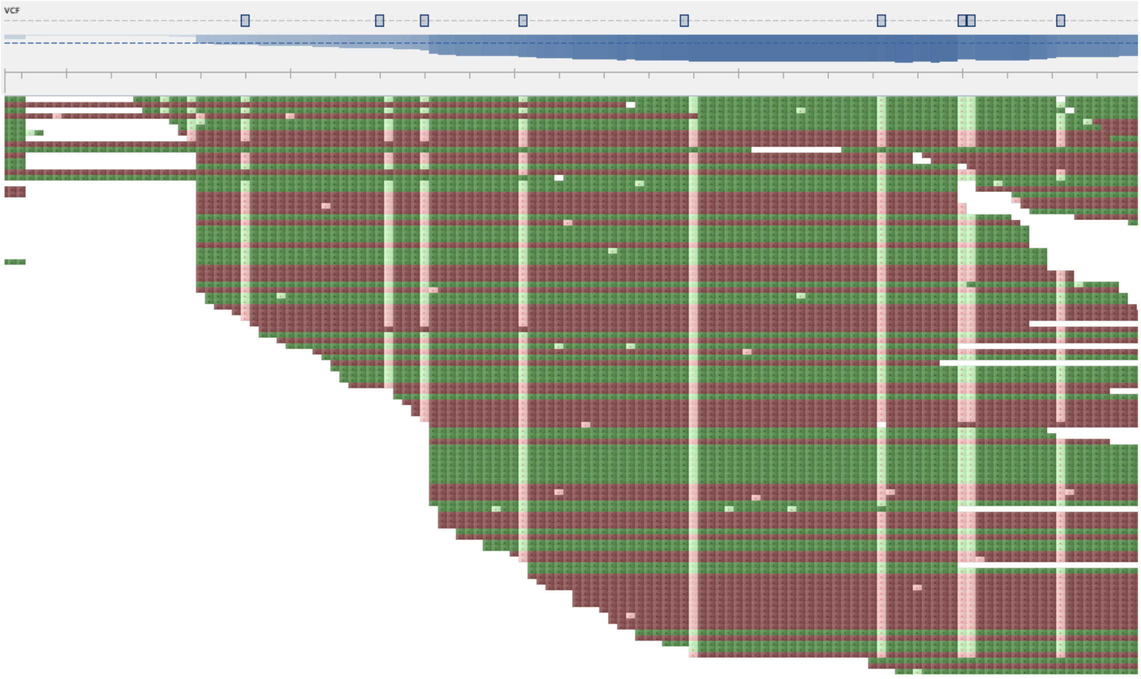

Fig. S19: Pileup visualization of SRR833154 reads aligned to a H37Rv graph using Tablet [5], with variant call annotations included on top.

20 CHOP, String graphs, and de Bruijn graphs

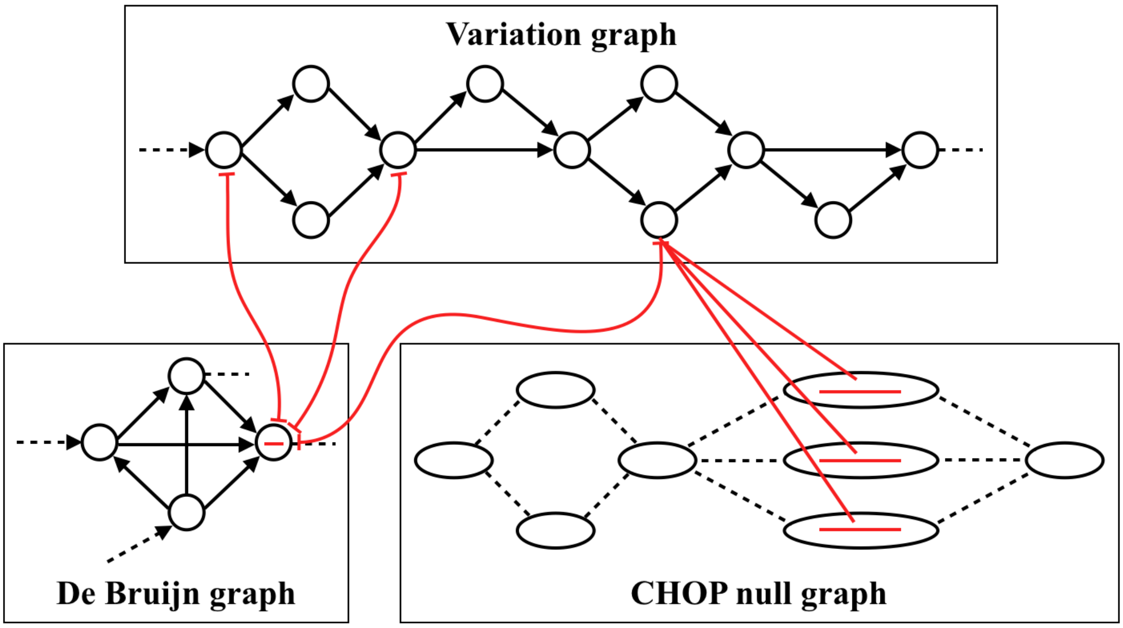

Fig. S20: Multiple positions in the variation graph can map to the same position in a de Bruijn graph index, while CHOP ensures an unique mapping for each.

21 Pseudocode CHOP procedures

```
procedure ChopGraph(G)
    SimplifyGraph(G)                              ▷ Extend and Collapse until exhaustion
    if G_E ≠ ∅ then
        for each edge (u, v) ∈ G_E do
            if deg(u) > deg(v) then
                Duplicate(G, u)                   ▷ Duplicate u
            else
                Duplicate(G, v)                   ▷ Duplicate v
        ChopGraph(G)

procedure SimplifyGraph(G)
    modified ← True
    while modified do
        modified ← False
        for each edge (u, v) ∈ G_E do
            if out(u) = 1 and in(v) = 1 then
                Collapse(G, u, v)                 ▷ Collapse u||v
                modified ← True
            else if in(v) = 1 and |u_S| ≥ k − 1 then
                Extend(G, u, v, 1)                ▷ Prefix u ↠ v
                modified ← True
            else if out(u) = 1 and |v_S| ≥ k − 1 then
                Extend(G, u, v, 0)                ▷ Suffix v ↞ u
                modified ← True
```

Listing S1:

```
procedure Collapse(G, u, v)
    if in(u) > out(v) then                        ▷ u ← v
        u_S = u_S · · · v_S                       ▷ Concatenate sequence
        for each edge (v, x) ∈ G_E do             ▷ Outgoing edges u
            add edge (u, x)
        delete node v
    else                                          ▷ u → v
        v_S = u_S · · · v_S                       ▷ Concatenate sequence
        for each edge (x, u) ∈ G_E do             ▷ Incoming edges v
            add edge (x, v)
        delete node u
```

Listing S2:

```
procedure Extend(G, u, v, isPrefix)
    if isPrefix then                                    ▷ u ↠ v
        v_S = u_S [|u_S| − k − 1, |u_S|] · · · v_S
    else                                                ▷ v ↞ u
        u_S = u_S · · · v_S [0, k − 1]
    delete edge (u, v)
```

Listing S3:

```
procedure Duplicate(G, u)
    for each edge (p, u) ∈ predecessors (G, u) do
        for each edge (u, s) ∈ successors (G, u) do
            group ← (x, u)_H ∩ (u, x)_H
            if group ≠ ∅ then
                create node i                           ▷ i ← u
                create edges ([(p, i), (i, s)])
    delete node u
```

Listing S4:

References

[1] Stephen F Altschul et al. Basic local alignment search tool. *Journal of molecular biology*, 215(3):403–410, 1990.

[2] Manuel Holtgrewe. Mason–a read simulator for second generation sequencing data. *Technical Report FU Berlin*, 2010.

[3] Daehwan Kim et al. Graph-based genome alignment and genotyping with HISAT2 and HISAT-genotype. *Nature biotechnology*, 37(8):907–915, 2019.

[4] Jasper Linthorst et al. Scalable multi whole-genome alignment using recursive exact matching. *BioRxiv*, p. 022715, 2015.

[5] Iain Milne et al. Tablet-next generation sequence assembly visualization. *Bioinformatics*, 26(3):401–402, 2009.

[6] Jacob Pritt et al. FORGe: prioritizing variants for graph genomes. *Genome biology*, 19(1):220, 2018.

[7] Mikko Rautiainen et al. Bit-parallel sequence-to-graph alignment. *Bioinformatics*, 03 2019.

[8] Jouni Sirén et al. Indexing graphs for path queries with applications in genome research. *Computational Biology and Bioinformatics, IEEE/ACM Transactions on*, 11(2):375–388, 2014.
